# FDX1 is associated with CALR abundance and JAK–STAT-related inflammatory signaling in myeloproliferative neoplasms

**DOI:** 10.3389/fonc.2026.1941311

**Published:** 2026-09-28

**Authors:** Diyaer Abuduaini, Diernu Simayi, Mihelayi Maimaiti, Yinglan Feng, Yixia Wang

**Affiliations:** Department of Hematology, The Second People’s Hospital of Kashgar, Kashgar, China

**Keywords:** bone marrow microenvironment, CALR, cuproptosis-related metabolism, FDX1, single-cell RNA-seq

## Abstract

**Introduction:**

Myeloproliferative neoplasms (MPNs) are driven by constitutive cytokine-receptor signaling, yet the metabolic programs associated with driver genotype and inflammatory remodeling remain incompletely defined.

**Methods:**

Here we integrated a 60-case clinical MPN cohort, the GSE174060 Affymetrix Human Transcriptome Array 2.0 microarray dataset, immune deconvolution, exploratory feature prioritization, the GSE228995 mouse single-cell RNA sequencing (scRNA-seq) dataset, human-sample validation, and functional perturbation assays to investigate whether cuproptosis-related metabolism is associated with total calreticulin (CALR) abundance and Janus kinase–signal transducer and activator of transcription (JAK–STAT)-related inflammatory signaling. GSE174060 included 36 samples: 6 essential thrombocythemia (ET), 11 polycythemia vera (PV), 9 primary myelofibrosis (PMF), 4 secondary myelofibrosis (SMF), and 6 healthy control (HC) samples.

**Results:**

Clinically, CALR-mutated cases were enriched for thrombocytosis and distinctive megakaryocytic abnormalities, while inflammatory and fibrotic features tracked with higher interleukin-6 (IL-6), tumor necrosis factor-α (TNF-α), and lactate dehydrogenase (LDH). Microarray-based transcriptomic analyses identified an MPN-associated cuproptosis-related and immune-response program, and consensus clustering separated samples into exploratory transcriptomic clusters. Within this expression-derived partition, *post hoc* multi-algorithm feature prioritization recurrently retained FDX1 and CALR among genes associated with the Cluster 1-versus-Cluster 2 transcriptional contrast. Single-cell analyses localized Calr and Fdx1 across hematopoietic and immune populations and highlighted inferred JAK–STAT-associated communication potential. In human MPN samples, FDX1 and total CALR protein and mRNA levels were increased and accompanied by STAT5 phosphorylation and elevated inflammatory mediators. Functionally, FDX1 knockdown reduced total CALR abundance, STAT5 phosphorylation, cytokine release, sensitivity to copper-dependent cytotoxicity, and proliferation, whereas FDX1 overexpression showed the reciprocal pattern and was partly counteracted by ruxolitinib.

**Discussion:**

These findings support an association between FDX1 perturbation, total CALR abundance, STAT5-associated signaling, and inflammatory output in MPN, but do not establish direct regulation of mutant CALR or mutant CALR–MPL signaling.

## Introduction

Philadelphia chromosome-negative myeloproliferative neoplasms (MPNs), including polycythemia vera (PV), essential thrombocythemia (ET), and primary myelofibrosis (PMF), are clonal hematopoietic disorders characterized by excessive myeloid production, thrombohemorrhagic risk and variable progression to marrow fibrosis or acute leukemia. Their contemporary classification integrates morphology, clinical phenotype and recurrent molecular lesions, with JAK2, CALR and MPL mutations forming the dominant driver framework (1, 2). The discovery of JAK2 V617F established constitutive Janus kinase–signal transducer and activator of transcription (JAK–STAT) signaling as a central mechanism in MPN pathogenesis, and subsequent studies identified CALR and MPL lesions as alternative routes into the same cytokine-receptor signaling network (3–7).

Among these drivers, CALR-mutated MPN represents a biologically distinctive state. CALR mutations are particularly enriched in JAK2-unmutated essential thrombocythemia and myelofibrosis, and mutant CALR can engage the thrombopoietin receptor MPL to induce downstream JAK–STAT activation (8–10). This receptor-level model explains much of the proliferative phenotype, but it does not fully account for the heterogeneity of megakaryocyte morphology, fibrosis, inflammatory cytokine production and treatment response observed among patients with otherwise related driver lesions.

Inflammation is increasingly viewed as both a consequence and a modifier of MPN evolution. Cytokines and inflammatory circuits can support clonal expansion, symptom burden, stromal remodeling and marrow fibrosis, while cytokine profiling in myelofibrosis has linked circulating inflammatory mediators to adverse clinical features and prognosis (10–13). JAK inhibition has transformed symptom control and spleen responses in myelofibrosis, yet it does not fully resolve clonal persistence or the upstream programs that couple mutant signaling to inflammatory and metabolic remodeling (14). These observations motivate a search for molecular intermediates that connect driver mutation status, immune-cell redistribution and disease-associated tissue remodeling. One candidate layer is cuproptosis-related metabolism. Cuproptosis is a copper-dependent form of cell death linked to lipoylated tricarboxylic-acid-cycle proteins, and ferredoxin 1 (FDX1) has emerged as a central regulator of this process (15). Although FDX1 has been explored as a cancer-associated cuproptosis-related gene and immunological correlate in pan-cancer analyses, its relationship to MPN driver signaling, total CALR abundance, CALR mutation status, and the hematopoietic microenvironment remains poorly defined (26). Because MPNs combine oncogenic cytokine signaling with profound changes in lineage output and inflammation, they provide a tractable setting in which to test whether a cuproptosis-associated program participates in driver-associated disease states.

In this study, we investigated the association among FDX1, total CALR abundance, CALR mutation status, and inflammatory JAK–STAT-associated signaling across complementary levels of evidence. Throughout this study, CALR mutation status refers to the presence or absence of a pathogenic CALR driver mutation, whereas CALR mRNA and protein measurements refer to total CALR abundance and do not distinguish mutant from wild-type CALR. We first mapped clinical, histopathological, and cytokine features in a 60-case MPN cohort, then integrated the GSE174060 gene-expression microarray dataset, CIBERSORT immune deconvolution, *post hoc* exploratory feature prioritization within the expression-derived transcriptomic partition, and GSE228995 single-cell RNA-seq communication analysis to identify candidate molecular relationships (16, 17). We further validated FDX1 and total CALR expression in human samples and tested the functional consequences of FDX1 perturbation in cellular models. Together, these analyses evaluate an FDX1-associated regulatory relationship with total CALR abundance, STAT5-associated signaling, cytokine release, and copper-dependent cytotoxicity in MPN.

## Materials and methods

### Study design

This study integrated clinical cohort analysis, public gene-expression microarray analysis, single-cell RNA-seq analysis, immune-deconvolution analysis, exploratory feature prioritization, cell–cell communication analysis, and experimental validation to evaluate associations among FDX1, total CALR abundance, CALR mutation status, cuproptosis-related programs, JAK–STAT-associated signaling, and inflammatory remodeling in MPN. The primary analytical workflow moved from clinical phenotype mapping, to transcriptomic discovery, to single-cell localization, and finally to human-sample and cell-model validation.

### Clinical cohort and phenotype annotation

A clinical cohort of 60 MPN cases was curated with disease subtype, driver mutation status, risk stratum, fibrosis grade, splenomegaly, systemic symptoms, bleeding history/acquired von Willebrand disease, peripheral blood counts, LDH, IL-6, TNF-α, bone marrow morphology, cytogenetics, treatment category, progression status, survival status, and follow-up time. MPN subtypes included PV, ET, prefibrotic primary myelofibrosis (prePMF), and overt PMF. Driver categories were JAK2, CALR, MPL, and triple-negative. For CALR-focused comparisons, cases were grouped as CALR-mutated versus non-CALR. Because the non-CALR group may include JAK2-mutated, MPL-mutated, triple-negative, and different MPN subtype contexts, CALR-versus-non-CALR comparisons should be interpreted as cohort-level associations unless adjusted, matched, or subtype-stratified analyses are provided.

### Ethics approval and consent to participate

This study was reviewed and approved by the Ethics Review Committee of The Second People’s Hospital of Kashgar (approval no. KSEY-EC-SOP/15/2.0-AF05; approval date: March 6, 2026). The approved project title was ‘Study on bone marrow histopathological morphology and immune regulation of the tumor microenvironment corresponding to different driver-gene mutation profiles in patients with myeloproliferative neoplasms.’ Written informed consent was obtained from participants for the collection and analysis of clinical information and human biological samples, including bone marrow and/or peripheral blood, according to the approved protocol.

### GSE174060 microarray dataset and preprocessing

GSE174060 is an expression-profiling-by-array dataset generated on the Affymetrix Human Transcriptome Array 2.0 platform. The official Gene Expression Omnibus (GEO) record contains 36 samples, including 6 ET, 11 PV, 9 PMF, 4 secondary myelofibrosis (SMF), and 6 healthy control (HC) samples (18). The original files are CEL files rather than RNA-seq raw-count files. For the analyses in this manuscript, the normalized gene-expression microarray matrix was imported into R, duplicate gene symbols were collapsed by retaining the row with the highest mean expression, and sample annotations were merged with subtype and disease-group labels. HC samples were treated as controls, whereas ET, PV, PMF, and SMF samples were grouped as MPN for disease-versus-control analyses.

### Principal component analysis and differential expression

For principal component analysis, the 1,000 genes with the highest variance were selected and scaled before principal component analysis (PCA). Differential expression between MPN and control samples was analyzed as microarray expression data using limma-style empirical Bayes moderated statistics on the normalized expression matrix (19). Genes were considered differentially expressed when the adjusted P value was < 0.05 and the absolute log2 fold change was > 0.2. Volcano plots displayed upregulated, downregulated, and non-significant genes, with core genes annotated where present.

### Core gene panel and cuproptosis-related score

The core gene panel included FDX1, CALR, JAK2, MPL, NFE2, SLC31A1, LIAS, and DLAT. The cuproptosis-related score was calculated as an unweighted mean expression score from available cuproptosis-associated genes in the normalized expression matrix, including FDX1, LIAS, LIPT1, DLD, DLAT, PDHA1, PDHB, MTF1, SLC31A1, ATP7A, and ATP7B. Because the score was not directionally weighted, it was used as a descriptive cuproptosis-associated expression summary rather than a validated measure of cuproptosis activity. Core gene expression and the cuproptosis-related score were compared across subtypes using Kruskal-Wallis tests. Heatmaps were generated from z-scored expression values after row-wise scaling.

### Functional enrichment analysis

Gene Ontology (GO) and Kyoto Encyclopedia of Genes and Genomes (KEGG) enrichment results were generated from MPN-associated differentially expressed genes and cluster-associated differentially expressed genes (20). GO terms were summarized by ontology category, including biological process, cellular component, and molecular function. Enrichment plots displayed term description, gene count, gene ratio where available, and adjusted P value or equivalent multiple-testing corrected score.

### Consensus clustering and exploratory transcriptomic cluster analysis

Consensus clustering was used to stratify MPN samples based on expression features (21). Consensus clustering was performed as an unsupervised approach to stratify MPN samples according to transcriptomic similarity, without using clinical subtype, driver mutation, fibrosis grade, or other clinical variables to define cluster membership. Candidate solutions from k = 2 to k = 5 were evaluated using consensus matrices, the consensus cumulative distribution function (CDF) and the relative change in area under the CDF curve. The k = 2 solution showed the clearest stable two-block structure and was therefore selected for downstream analyses. Because these clusters were transcriptomically derived and were not independently validated, they are referred to as exploratory transcriptomic clusters rather than clinically defined or validated molecular subtypes.

### Immune infiltration and gene-immune association analyses

Cell-type Identification By Estimating Relative Subsets Of RNA Transcripts (CIBERSORT) results were used to estimate immune-cell fractions from the bulk expression matrix (17). Major immune-cell populations were displayed as stacked bar plots and subtype-level heatmaps. Control-versus-MPN immune-cell comparisons were performed with Wilcoxon rank-sum tests, and multiple testing was controlled using Benjamini-Hochberg adjustment where indicated. Spearman correlation was used to assess co-expression among core genes and to associate FDX1, CALR, JAK2, MPL, NFE2, and the cuproptosis-related score with immune-cell fractions.

### Machine-learning feature selection

Machine-learning analysis was performed as a *post hoc* exploratory feature-prioritization procedure within the two expression-derived transcriptomic clusters. Cluster membership was represented as a binary variable. For LASSO and XGBoost, the two cluster labels were encoded as 0/1 and analyzed using binomial or binary-logistic objectives. In the existing random-forest and SVM-RFE workflows, the same cluster labels were supplied as numeric 0/1 values; consequently, these two analyses operated in regression mode and were used only for feature ranking with respect to the numerical representation of Cluster 1/Cluster 2 membership, rather than as binary classifiers. Genes with a Benjamini–Hochberg-adjusted P value < 0.05 and an absolute log2 fold change > 0.2 were included in the initial feature space. The same DEG-derived expression matrix was used as the input for least absolute shrinkage and selection operator (LASSO), random forest, support vector machine-recursive feature elimination (SVM-RFE), and extreme gradient boosting (XGBoost), thereby ensuring that differences among the selected feature sets reflected the algorithmic procedures rather than differences in the initial candidate-gene pool (22). LASSO analysis was performed using the glmnet R package with a binomial model and α = 1. Cross-validation was used to optimize the regularization parameter, and lambda.min, corresponding to the minimum cross-validated deviance, was selected. Genes with non-zero coefficients at lambda.min were retained as LASSO-selected features. Random forest analysis was performed using the randomForest R package with 500 trees. Because the response was supplied as numeric 0/1, the random forest was implemented in regression mode. Variable importance was evaluated using %IncMSE, representing the increase in prediction mean squared error after permutation of each feature, together with IncNodePurity as a complementary regression-tree importance measure. The resulting ranking was used only for exploratory feature prioritization and was not interpreted as classification accuracy or diagnostic performance. SVM-based feature selection was performed using recursive feature elimination with a radial-basis support vector machine. Because the Cluster 1/Cluster 2 outcome was supplied numerically, this workflow was likewise evaluated as a regression-based ranking procedure. Candidate feature subsets were assessed by cross-validated RMSE, and the subset associated with the minimum RMSE was retained for exploratory feature prioritization. In contrast, XGBoost was implemented as a binary classification model using a binary-logistic objective, with Cluster 1/Cluster 2 encoded as 0/1. The model used a learning rate (eta) of 0.5, a maximum tree depth of 2, and 10 boosting rounds, with AUC used as the evaluation metric. Feature importance was extracted using Gain, Cover, and Frequency, with Gain used as the primary ranking metric; genes with non-zero Gain were retained as XGBoost-selected features. RMSE was therefore not interpreted as a classification-performance metric. The complete feature sets obtained from LASSO, random forest, SVM-RFE, and XGBoost were subsequently compared using a Venn diagram. Genes retained by all four algorithms were defined as shared exploratory candidate features. Complete initial and algorithm-specific feature lists, together with the corresponding coefficients or importance measures, are provided in the **Supplementary Materials**. Because all four approaches were applied to the same dataset, this analysis was interpreted as multi-algorithm exploratory feature prioritization rather than independent model validation or evidence of biological causality (**Supplementary Materials**).

### GSE228995 single-cell RNA sequencing processing and clustering

GSE228995 is a Mus musculus 10x Genomics scRNA-seq dataset from a murine MPL W515L-induced myelofibrosis model. The official GEO record contains 22 samples (23), including mononuclear cells, stromal cells, CD41-positive cells and lineage-negative, c-Kit-positive (LK) hematopoietic stem and progenitor cell (HSPC)-enriched populations. In this manuscript, the analyzed subset was represented by five green fluorescent protein (GFP)-control samples and five MPL W515L model samples, displayed as GFP1-GFP5 and MPL1-MPL5. These labels were retained from the project analysis files. Because different sorted populations can introduce batch and cell-composition effects, the single-cell analysis was interpreted as exploratory. Quality control included visualization of detected genes per cell, total RNA counts, and mitochondrial-read percentage. The relationship between nFeature_RNA and nCount_RNA was assessed by scatter plot. The 2,000 most variable genes were selected for downstream analysis. Principal component analysis (PCA), elbow-plot inspection, graph-based clustering, clustree assessment across resolutions, and uniform manifold approximation and projection (UMAP) visualization were used to define cell clusters (24).

### Cell-type annotation and single-cell gene expression

Cell clusters were annotated using lineage marker genes displayed by dot plot. The annotated populations included B cells, bone marrow (BM) cells, erythroblasts, HSC_G-CSF cells, macrophages, monocytes, neutrophils, natural killer (NK) cells, Pre-B_cell_CD34-, and T cells. Calr and Fdx1 expression was visualized using UMAP feature plots, violin plots, and dot plots to determine their distribution across annotated cell types.

### Cell–cell communication analysis

Intercellular communication among annotated single-cell populations was inferred using a CellChat-style ligand-receptor framework (16). Circle plots summarized inferred interaction number and interaction strength among cell populations. JAK–STAT-associated communication probabilities were visualized as heatmaps, and network-role analysis was used to identify sender, receiver, mediator, and influencer states. Ligand-receptor bubble plots displayed selected interaction pairs, communication probability, and statistical significance. These analyses infer communication potential from ligand-receptor expression and do not directly measure pathway activation or physical cell–cell interactions.

### Human-sample validation

FDX1, total CALR, JAK2/STAT5 signaling proteins, inflammatory transcripts, and secreted cytokines were evaluated in control and MPN samples. Western blotting was used to assess FDX1, total CALR, phosphorylated JAK2 (p-JAK2), total JAK2, phosphorylated STAT5 (p-STAT5), total STAT5, and β-actin. Quantitative polymerase chain reaction (qPCR) was used to quantify FDX1, CALR, IL6, JAK2, MPL, MYC, STAT5, and VEGFA. Enzyme-linked immunosorbent assay (ELISA) was used to measure interleukin-6 (IL-6) and transforming growth factor-β1 (TGF-β1). Protein and mRNA values were normalized to the indicated internal controls and expressed relative to the control group. CALR mutation status was analyzed as a genetic/clinical variable. In contrast, CALR expression measured by qPCR and Western blotting represents total CALR mRNA or protein abundance. These assays were not allele-specific and therefore did not distinguish mutant CALR from wild-type CALR. Accordingly, total CALR abundance was analyzed separately from CALR mutation status and was not interpreted as a direct measure of mutant CALR abundance, mutant CALR activity, or mutant CALR–MPL signaling.

### Cell culture and perturbation experiments

Peripheral blood mononuclear cells (PBMCs), SET-2 cells, and CALR-mutant cells were used for cell-based validation. FDX1 was silenced using small interfering RNA (siRNA), and rescue experiments were performed by reintroducing FDX1 in the knockdown background. FDX1-overexpression experiments were performed with vector control and FDX1-overexpression conditions, with ruxolitinib treatment used to test JAK–STAT pathway dependence. Knockdown, overexpression, and rescue effects were evaluated by western blotting for FDX1, total CALR, p-STAT5, and β-actin.

### Immunofluorescence

Cells from negative-control small interfering RNA (siNC), siFDX1, and siFDX1-rescue conditions were fixed, stained for p-STAT5, counterstained with 4′,6-diamidino-2-phenylindole (DAPI), and imaged by fluorescence microscopy. p-STAT5 intensity was quantified from fluorescence images and compared among groups.

### ELISA and cell-viability assays

Culture supernatants from siNC, siFDX1, and siFDX1-rescue conditions were analyzed by ELISA for IL-6, IL-8, and TGF-β1. Copper-dependent cytotoxicity was assessed after treatment with dimethyl sulfoxide (DMSO), CuCl_2_, elesclomol plus CuCl_2_, elesclomol plus CuCl_2_ with siFDX1, or elesclomol plus CuCl_2_ with tetrathiomolybdate (TTM). Viability was reported as percentage of the DMSO control. Cell proliferation was measured by Cell Counting Kit-8 (CCK-8) and reported as optical density at 450 nm (OD450) at 24, 48, and 72 h in siNC, siFDX1, and siFDX1-rescue groups.

### Statistical analysis

Statistical analyses were performed in R or the corresponding plotting workflow. Wilcoxon rank-sum tests were used for two-group clinical and immune-cell comparisons. Spearman correlation was used for fibrosis-grade associations, gene co-expression, and gene-immune association analyses. Kruskal-Wallis tests were used for subtype-level comparisons across more than two groups. Welch t-tests were used for displayed CCK-8/copper-dependent cytotoxicity comparisons where specified by the plotting script. P < 0.05 was considered statistically significant unless otherwise stated; multiple testing was adjusted by the Benjamini-Hochberg method where indicated.

### Data management and reproducibility

All analyses were performed using the figure-associated source tables and analysis outputs retained in the project archive. Public transcriptomic datasets were analyzed from their original repository records, and clinical and experimental data were checked against the corresponding source files before figure generation. **Supplementary Data** provides supporting tables for antibodies, qPCR primers, perturbation reagents, key assay reagents, software workflows, datasets, and recommended source-data files.

## Results

### Clinical cohort profiling showed that CALR-mutated MPN was linked to thrombocytosis, megakaryocyte dysplasia, and fibrosis-associated inflammation

We first defined the clinical and genetic structure of the 60-case MPN cohort. Driver mutation mapping showed that JAK2 dominated across PV, ET, prePMF, and overt PMF, whereas CALR mutations were concentrated in ET and PMF-related disease, with smaller MPL-mutated and triple-negative subsets (**Figure 1a**). A patient-level phenotype map further showed that MPN subtype, driver status, risk group, fibrosis grade, splenomegaly, systemic symptoms, and bleeding/acquired von Willebrand disease varied together across the cohort rather than forming isolated clinical features (**Figure 1b**).

**Figure 1:**
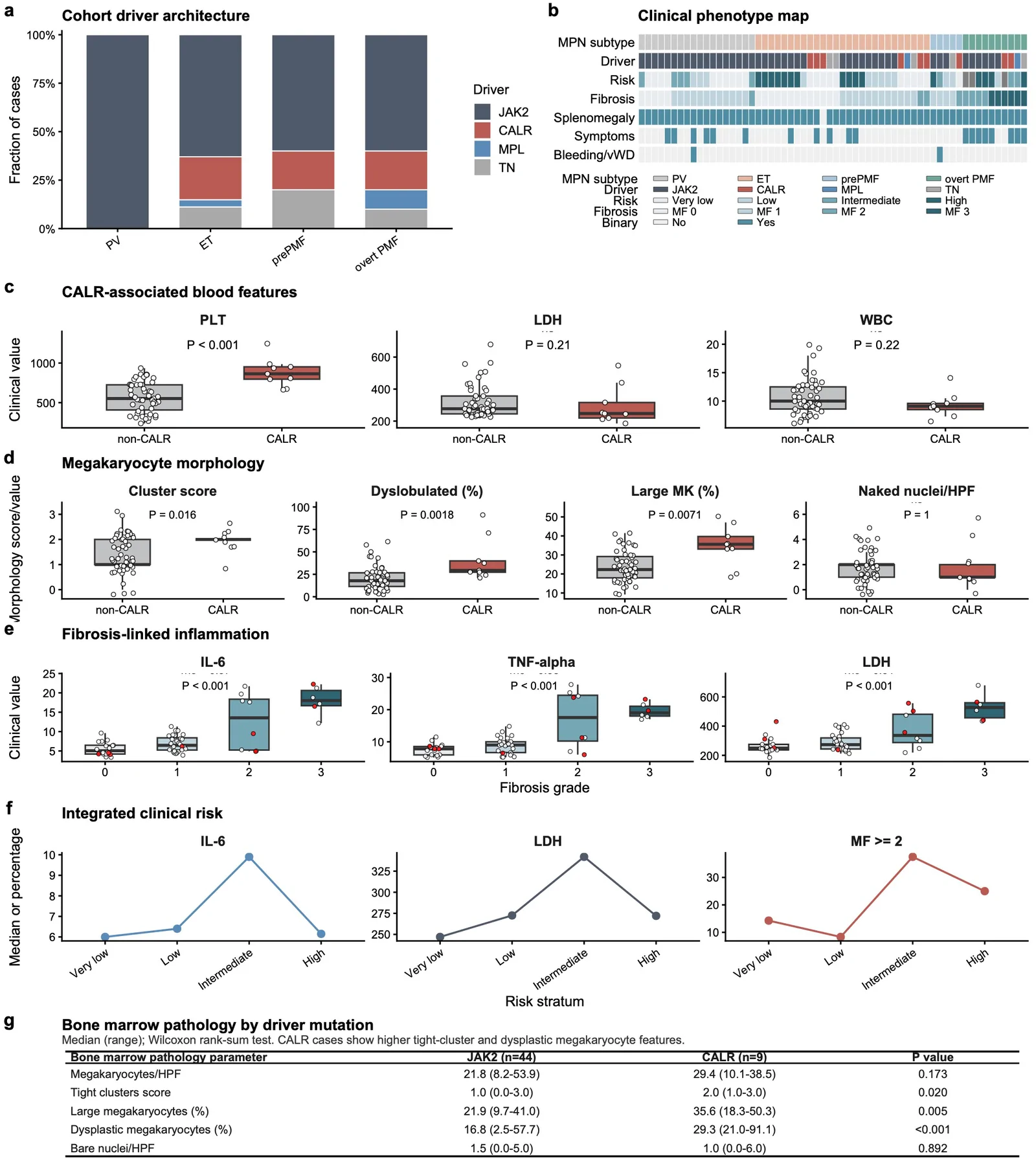
Clinical architecture of the myeloproliferative neoplasm (MPN) cohort and calreticulin (CALR)-associated clinicopathologic features. **(a)** Driver mutation composition across polycythemia vera (PV), essential thrombocythemia (ET), prefibrotic primary myelofibrosis (prePMF), and overt primary myelofibrosis (PMF) cases. **(b)** Patient-level clinical phenotype map showing MPN subtype, driver mutation, risk stratum, fibrosis grade, splenomegaly, systemic symptoms, and bleeding/acquired von Willebrand disease. **(c)** CALR-mutated cases showed higher platelet counts than non-CALR cases, while lactate dehydrogenase (LDH) and white blood cell (WBC) count did not differ significantly. **(d)** CALR-mutated cases showed higher tight-cluster score, dyslobulated megakaryocyte ratio, and large megakaryocyte ratio, with no difference in naked nuclei per high-power field. **(e)** Interleukin-6 (IL-6), tumor necrosis factor-α (TNF-α), and LDH increased with fibrosis grade. **(f)** Integrated clinical risk strata summarized median IL-6, median LDH, and the percentage of cases with myelofibrosis (MF) grade ≥2. **(g)** Bone marrow pathology comparison between JAK2- and CALR-mutated cases. Wilcoxon rank-sum tests were used for group comparisons, and Spearman correlation was used for fibrosis-linked associations where applicable.

After establishing this cohort structure, we compared CALR-mutated cases with non-CALR cases to define the phenotype associated with CALR. CALR-mutated cases had significantly higher platelet counts (P < 0.001), whereas LDH and WBC did not differ significantly between CALR and non-CALR cases (P = 0.21 and P = 0.22, respectively; **Figure 1c**). Bone marrow morphology showed the same direction of CALR-associated remodeling: tight megakaryocyte cluster score, dyslobulated megakaryocyte percentage, and large megakaryocyte percentage were higher in CALR-mutated cases, while naked nuclei per high-power field was unchanged (**Figure 1d**).

We then asked whether the clinical phenotype was coupled to inflammatory and fibrotic severity. IL-6, TNF-α, and LDH increased with fibrosis grade, placing inflammatory activation alongside marrow fibrosis (**Figure 1e**). Integrated risk-stratum plots showed that IL-6, LDH, and the proportion of cases with MF grade ≥2 were highest or near highest in intermediate/high-risk strata, although the pattern was not strictly linear across all categories (**Figure 1f**). Finally, direct comparison of bone marrow pathology by driver mutation showed that CALR-mutated cases had higher tight-cluster scores, large megakaryocyte fractions, and dysplastic megakaryocyte fractions than JAK2-mutated cases, while megakaryocyte number and bare nuclei were not significantly different (**Figure 1g**). Together, **Figure 1** established the clinical basis for studying a CALR-linked inflammatory and megakaryocytic phenotype in MPN.

### GSE174060 microarray profiling identified an MPN-associated cuproptosis-related and immune-response program

We next analyzed the GSE174060 Affymetrix Human Transcriptome Array 2.0 microarray dataset, which comprised 6 HC, 6 ET, 11 PV, 9 PMF, and 4 SMF samples (**Figure 2a**). Principal component analysis of the 1,000 most variable genes separated most control and MPN samples, although some overlap remained (**Supplementary Figure 1a**). Differential-expression analysis identified upregulated and downregulated genes at an adjusted P value < 0.05 and |log2 fold change| > 0.2 (**Supplementary Figure 1b**).

**Figure 2:**
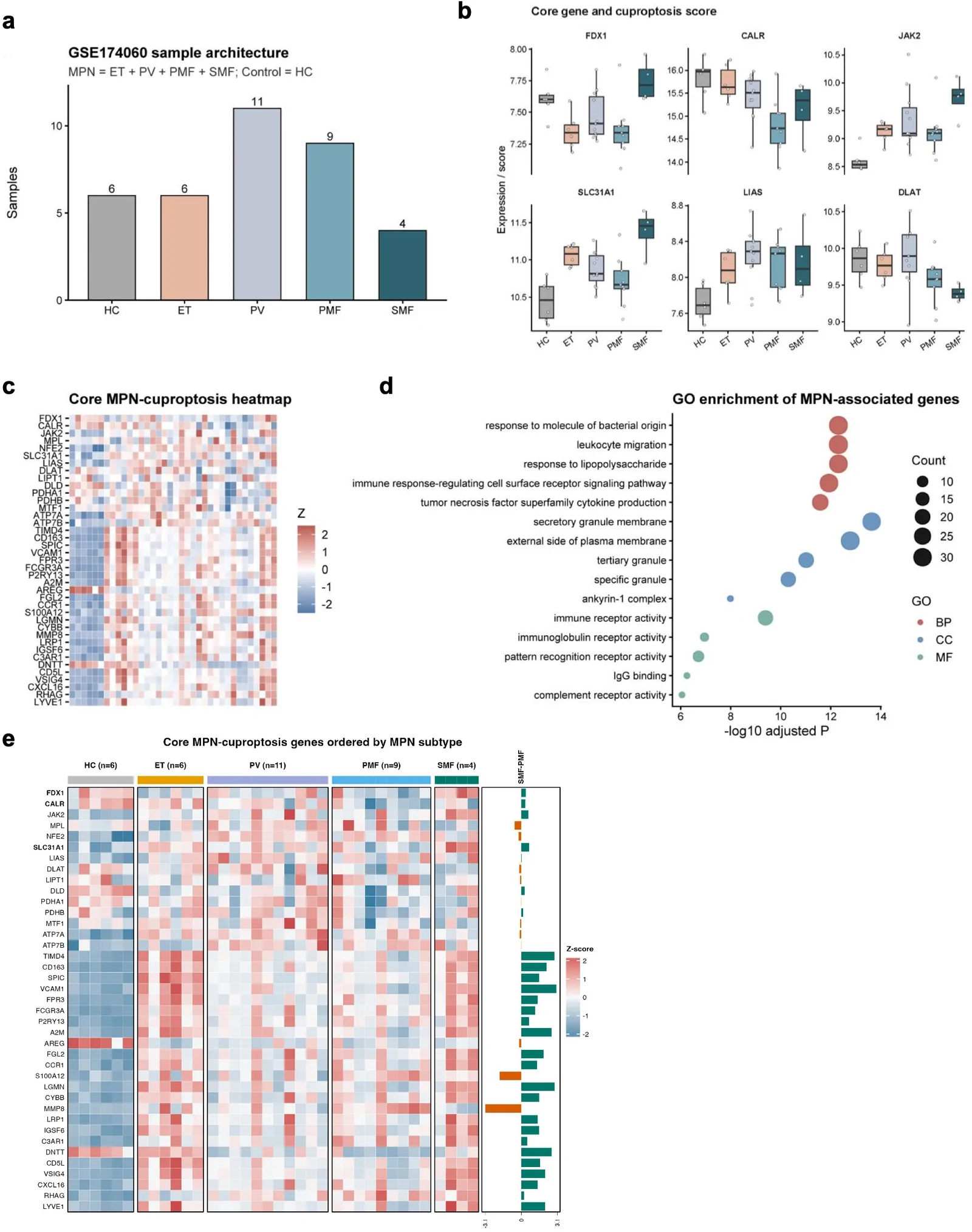
GSE174060 microarray profiling defines MPN-associated cuproptosis-related, immune, and subtype-level expression patterns. **(a)** GSE174060 sample composition, including 6 healthy control (HC), 6 ET, 11 PV, 9 PMF, and 4 secondary myelofibrosis (SMF) samples. **(b)** Expression of FDX1, CALR, JAK2, SLC31A1, LIAS, and DLAT across the five groups. **(c)** Heatmap of core MPN/cuproptosis-associated genes and highly ranked MPN-associated genes across individual samples. **(d)** Gene Ontology enrichment of MPN-associated genes; dot size indicates gene count, color denotes biological process, cellular component, or molecular function, and the x axis shows -log10 adjusted P. **(e)** Subtype-ordered heatmap of core MPN/cuproptosis-associated and immune-related genes; the right-hand bars summarize the SMF-minus-PMF contrast for each displayed gene. Heatmaps display row-wise z scores.

Subtype-level analyses showed variation in FDX1, CALR, JAK2, SLC31A1, LIAS, and DLAT expression across HC, ET, PV, PMF, and SMF samples (**Figure 2b**). Heatmaps showed heterogeneous core-gene and MPN-associated expression patterns across individual samples and disease subtypes (**Figures 2c, e**). Gene Ontology enrichment of MPN-associated genes highlighted responses to bacterial-origin molecules and lipopolysaccharide, leukocyte migration, immune receptor activity, and secretory-granule and plasma-membrane components (**Figure 2d**). When the core MPN/cuproptosis-associated genes were ordered by subtype, FDX1, CALR, and SLC31A1 showed higher relative expression in SMF than in PMF, with JAK2 showing a similar trend (**Figure 2e**). LIAS and DLD showed modest changes in the same direction, whereas DLAT, LIPT1, MTF1, ATP7A, MPL, and NFE2 did not display a uniform SMF-high pattern. Thus, the SMF–PMF difference appeared selective rather than reflecting pathway-wide upregulation. Because only four SMF samples were available, these observations were considered exploratory. In a complementary CALR-mutated versus non-CALR analysis, whole-transcriptome structure remained heterogeneous, and none of the eight displayed core genes passed a Benjamini-Hochberg false-discovery-rate threshold of 0.05 (**Supplementary Figures 2a-c**). Gene Ontology biological-process enrichment nevertheless identified programs that differed between these groups (**Supplementary Figure 2d**). Together, these analyses linked the MPN transcriptome to cuproptosis-related genes and immune remodeling while showing that CALR-associated core-gene differences were not significant in this exploratory comparison.

### Consensus clustering separated MPN samples into exploratory transcriptomic clusters with distinct immune and lineage programs

To determine whether the MPN transcriptomic landscape contained reproducible internal structure, we performed consensus clustering. Consensus matrices for k = 2 to k = 5, cumulative distribution function curves, and relative changes in area under the cumulative distribution function showed the clearest stable two-block structure at k = 2, with diminishing gains at higher k values (**Supplementary Figures 3a–f**). We therefore selected two exploratory transcriptomic clusters for downstream comparison.

The Cluster 1-versus-Cluster 2 volcano plot showed broad differential expression, with both upregulated and downregulated genes (**Figure 3a**). Several displayed driver and cuproptosis-related genes differed between clusters, including NFE2, JAK2, LIAS, FDX1, DLAT, CALR, and other myeloid-associated genes (**Figure 3b**). Kyoto Encyclopedia of Genes and Genomes enrichment linked cluster-associated genes to neutrophil extracellular trap formation, phagosome, hematopoietic cell lineage, complement and coagulation cascades, and acute myeloid leukemia-related pathways (**Figure 3c**). Gene Ontology enrichment highlighted immune-response-regulating receptor signaling, leukocyte migration, responses to bacterial-origin molecules and lipopolysaccharide, secretory-granule compartments, and immune receptor activity (**Figure 3d**). These results indicated that the exploratory clusters were associated with immune and lineage-remodeling programs.

**Figure 3:**
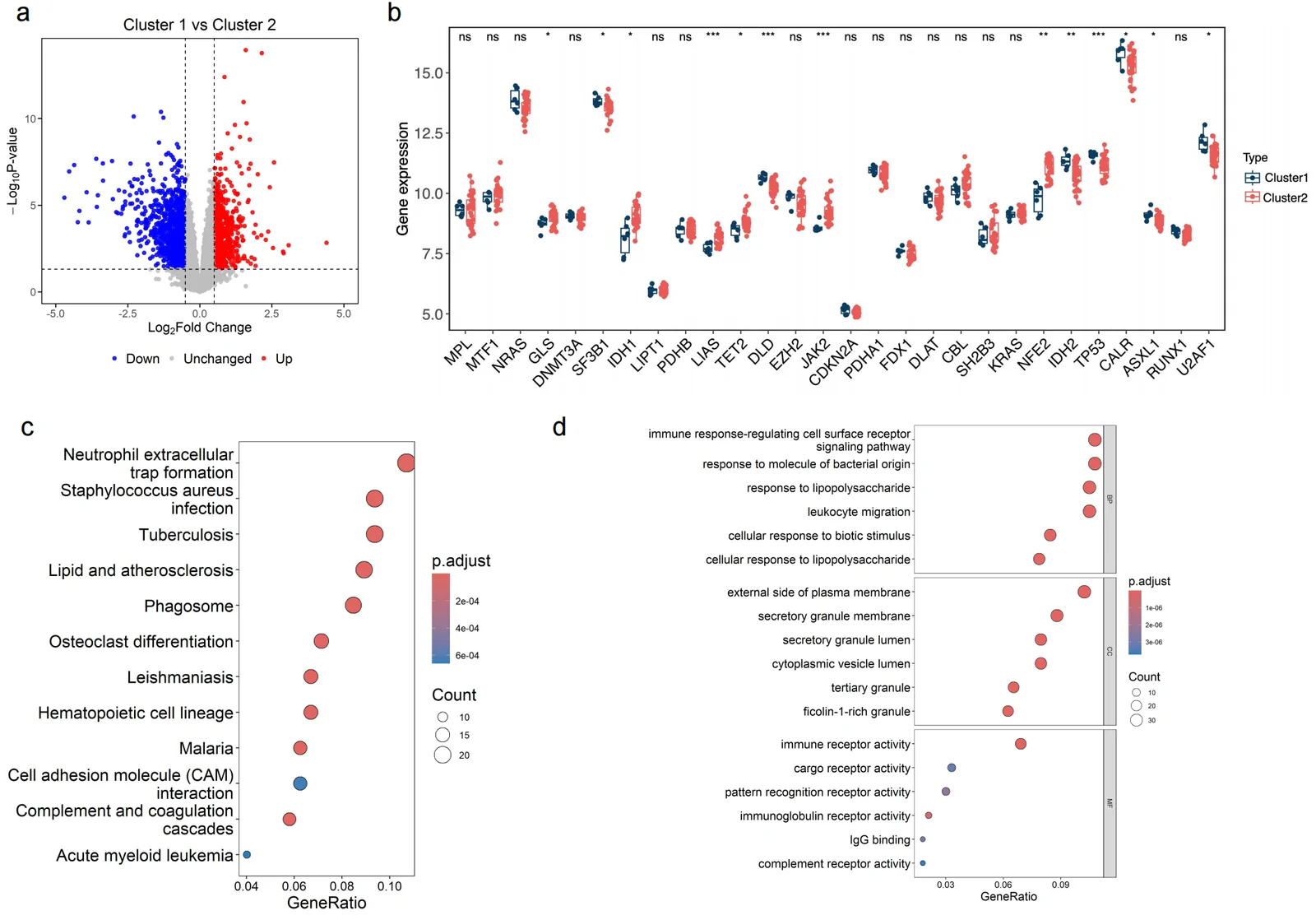
Differential-expression and functional-enrichment profiles of two exploratory MPN transcriptomic clusters. **(a)** Volcano plot comparing Cluster 1 with Cluster 2; red, blue, and gray points denote upregulated, downregulated, and unchanged genes, respectively. **(b)** Box-and-jitter plots of selected driver, cuproptosis-related, and myeloid-associated genes in the two clusters; ns, not significant. **(c)** Kyoto Encyclopedia of Genes and Genomes enrichment of cluster-associated genes; the x axis shows gene ratio, dot size indicates gene count, and color indicates adjusted P. **(d)** Gene Ontology enrichment across biological process, cellular component, and molecular function categories, with gene ratio, count, and adjusted P encoded as in panel c. Cluster labels denote exploratory transcriptomic groups and not validated clinical subtypes. *P < 0.05; **P < 0.01; ***P < 0.001; ns, not significant.

### Core MPN/cuproptosis-related genes were associated with immune-cell redistribution

Because the enrichment analyses repeatedly pointed toward immune remodeling, we next integrated the core gene panel with immune deconvolution. Spearman co-expression analysis showed positive relationships among several core genes, including a positive correlation between FDX1 and CALR, and stronger positive correlations between CALR and DLAT, JAK2 and SLC31A1, JAK2 and NFE2, JAK2 and LIAS, NFE2 and LIAS, and NFE2 with JAK2-related features (**Figure 4a**). CIBERSORT deconvolution revealed that individual samples contained variable proportions of monocytes, neutrophils, naive B cells, plasma cells, mast cells, eosinophils, NK cells, macrophages, and CD4 T-cell subsets (**Figure 4b**).

**Figure 4:**
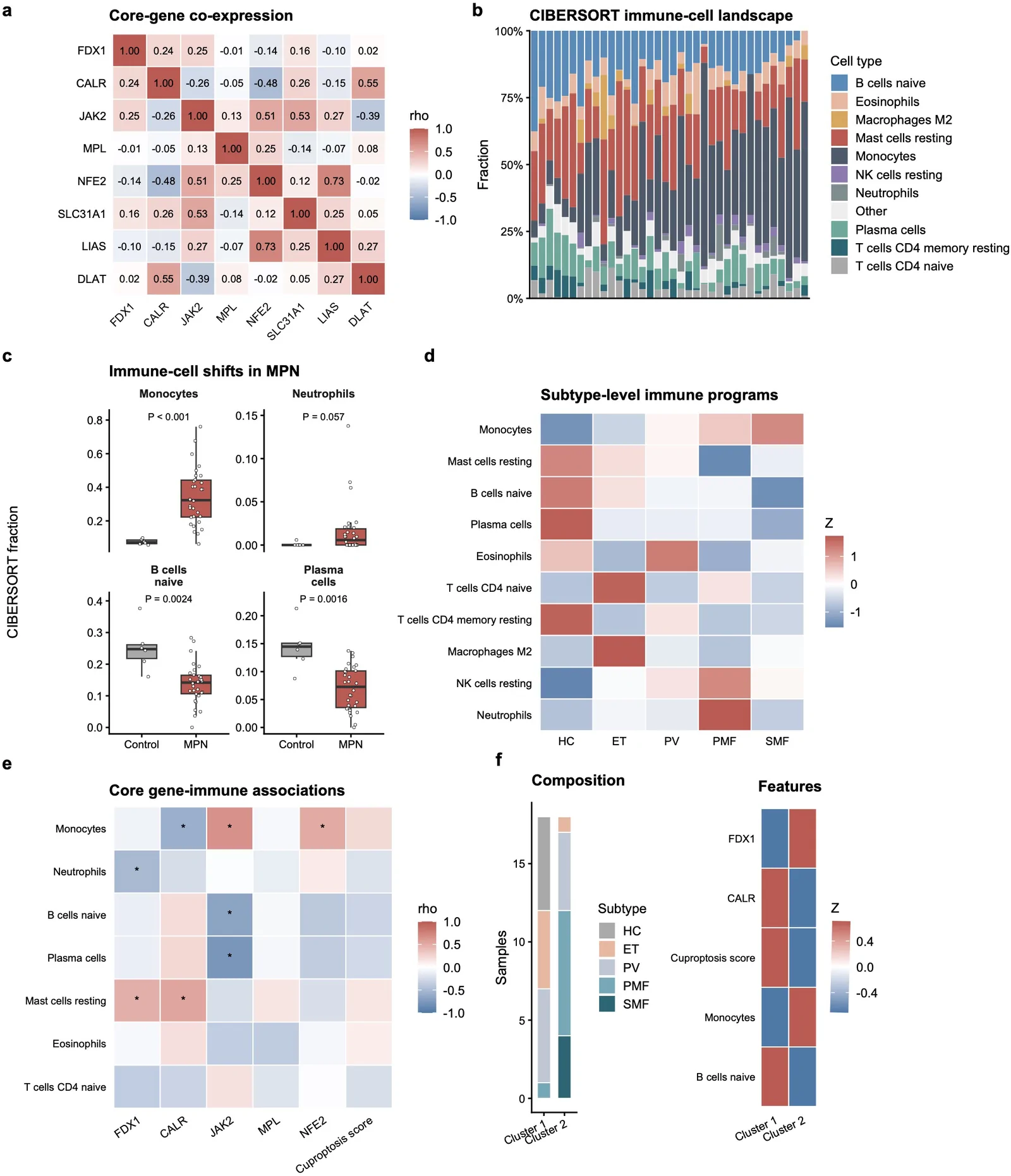
Core gene co-expression and immune-cell infiltration in MPN. **(a)** Spearman co-expression matrix for FDX1, CALR, JAK2, MPL, NFE2, SLC31A1, LIAS, and DLAT. **(b)** Cell-type Identification By Estimating Relative Subsets Of RNA Transcripts (CIBERSORT)-inferred relative immune-cell fractions across samples. **(c)** Immune-cell shifts between control and MPN samples, showing increased monocytes, decreased naive B cells and plasma cells, and a trend toward increased neutrophils. **(d)** Subtype-level immune-cell program heatmap. **(e)** Associations between core genes or cuproptosis-related score and immune-cell fractions; asterisks denote Benjamini-Hochberg-adjusted significance where indicated. **(f)** Integrated molecular-cluster and immune-feature summary.

Group-level comparison showed a clear disease-associated immune shift. Monocytes were significantly increased in MPN samples compared with controls (P < 0.001), naive B cells and plasma cells were significantly decreased (P = 0.0024 and P = 0.0016), and neutrophils showed an increasing trend (P = 0.057; **Figure 4c**). At the subtype level, HC, ET, PV, PMF, and SMF displayed distinct immune programs, indicating that immune composition differed across the MPN spectrum (**Figure 4d**).

We then asked whether this immune variation was related to the core gene panel. Gene-immune association heatmaps showed significant relationships between selected genes and immune-cell fractions after multiple-testing correction, including associations involving FDX1, CALR, JAK2, NFE2, and the cuproptosis-related score (**Figure 4e**). Finally, integration of Cluster 1/Cluster 2 composition with FDX1, CALR, cuproptosis-related score, monocytes, and naive B-cell features showed that the exploratory clusters differed in several immune-associated features (**Figure 4f**). These findings suggest that MPN-related expression patterns are coupled to relative immune-cell redistribution.

### *Post hoc* feature prioritization highlighted FDX1 and CALR within the exploratory transcriptomic partition

To rank genes associated with the two exploratory transcriptomic states, we performed a *post hoc* multi-algorithm analysis using the complete set of Cluster 1-versus-Cluster 2 differentially expressed genes as a common initial feature space. Because both cluster membership and the DEG feature space were derived from the same expression dataset, this analysis was intended to characterize and prioritize genes associated with the existing transcriptomic partition rather than to independently discover or validate classifiers of cluster membership. The same DEG-derived expression matrix was supplied to LASSO, random forest, SVM-RFE, and XGBoost, and algorithm-specific selection criteria were defined before calculating the intersection of the resulting feature sets. LASSO cross-validation identified lambda.min as the penalty parameter associated with the minimum cross-validated deviance, and genes with non-zero coefficients at this value were retained (**Figures 5a, b**). The regression-mode random-forest analysis using 500 trees ranked candidate genes according to %IncMSE and IncNodePurity, and the 20 highest-ranking genes by %IncMSE were retained (**Figures 5c, d**). The regression-based SVM-RFE analysis evaluated progressively reduced feature subsets by 10-fold cross-validation, with the subset producing the minimum RMSE selected as the optimal feature set (**Figure 5e**). XGBoost provided a complementary tree-based ranking, with Gain used as the primary importance metric and genes with non-zero Gain retained as candidate features (**Figure 5f**). Intersection of the four algorithm-derived feature sets recurrently retained FDX1 and CALR among genes associated with the Cluster 1-versus-Cluster 2 transcriptional contrast (**Figure 5g**; **Supplementary Materials**). Because the four procedures used the same samples, cluster labels, and DEG-derived feature space, this overlap represents cross-algorithm concordance within the same *post hoc* analysis and should not be interpreted as four independent lines of computational evidence (**Figure 5g**; **Supplementary Materials**). Gene set enrichment analysis further linked the ranked transcriptional state to immune-response activation (**Figure 5h**), while STRING-based interaction networks placed FDX1 and CALR within distinct protein-association neighborhoods (**Figure 5i**). These analyses were therefore used only for exploratory within-partition feature prioritization and not for independent biomarker discovery, validation of the transcriptomic clusters, or estimation of generalizable predictive performance.

**Figure 5:**
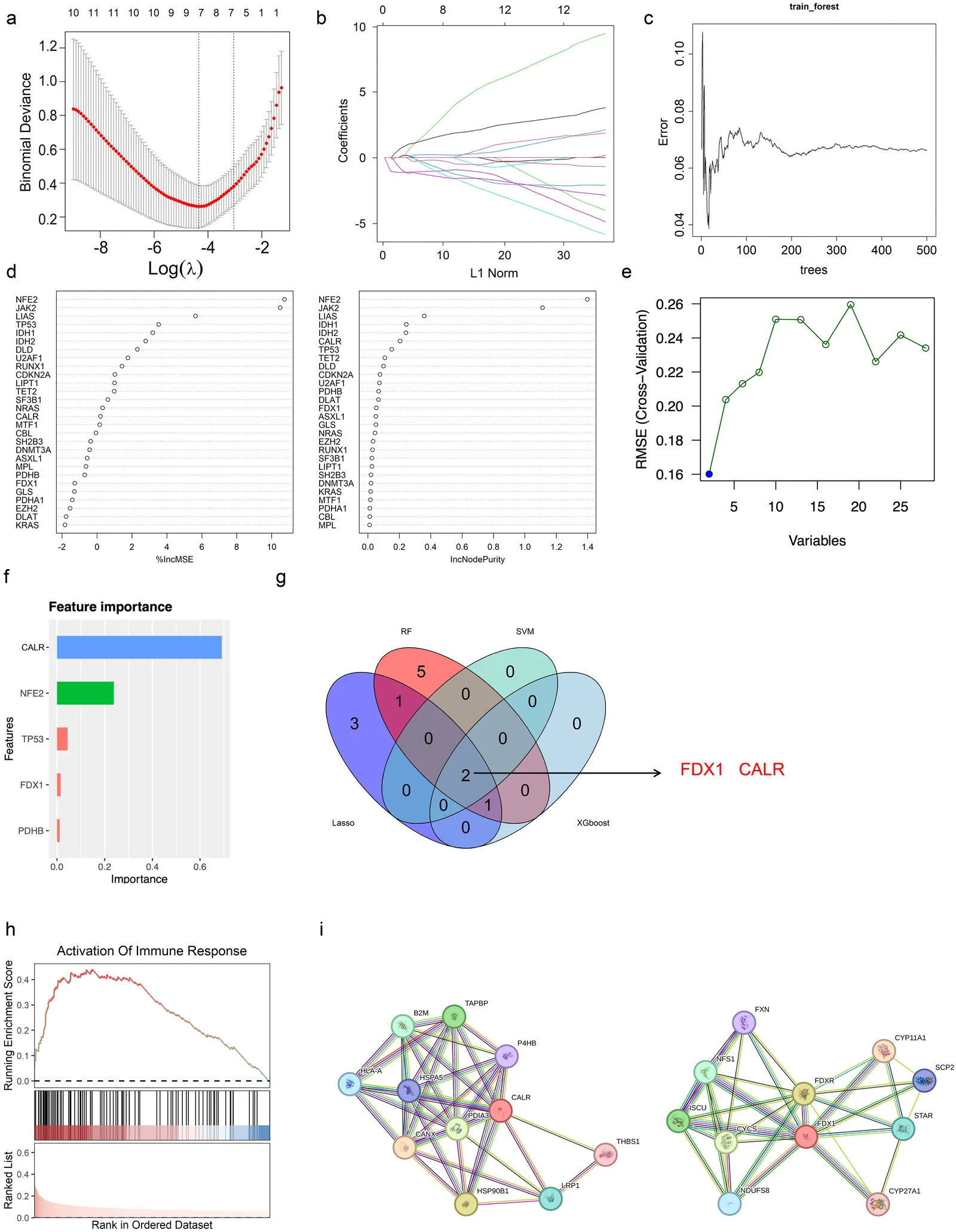
Exploratory machine-learning prioritization identifies FDX1 and CALR as shared candidate features. **(a)** Cross-validation curve for LASSO model selection. The vertical reference lines indicate lambda.min and lambda.1se; lambda.min was used for final feature selection. **(b)** LASSO coefficient paths for the DEG-derived candidate feature space. Genes with non-zero coefficients at lambda.min were retained.**(c)** Prediction error across 500 trees for the regression-mode random-forest analysis using the numeric 0/1 representation of Cluster 1/Cluster 2 membership.**(d)** Random-forest regression feature importance. %IncMSE denotes the increase in prediction mean squared error after feature permutation, and IncNodePurity denotes the cumulative contribution of a feature to reduction in regression-tree node impurity.**(e)** Regression-based SVM-RFE analysis showing cross-validated RMSE across candidate feature numbers; the feature subset associated with the minimum RMSE was retained for exploratory feature prioritization. **(f)** XGBoost binary-logistic feature-importance analysis ranked according to Gain. **(g)** Venn diagram showing the intersection of the complete LASSO-, random forest-, SVM-RFE-, and XGBoost-selected feature sets. **(h)** Gene set enrichment analysis (GSEA) showing immune-response activation in the ranked expression state. **(i)** STRING-derived interaction-neighborhood plots for FDX1 and CALR. Because Cluster 1/Cluster 2 labels and the DEG-derived feature space originated from the same expression dataset, these analyses represent *post hoc* feature prioritization within an expression-defined partition and do not constitute independent biomarker discovery, cluster validation, or unbiased estimation of predictive performance. LASSO and XGBoost used binary-response objectives, whereas the random-forest and SVM-RFE analyses used regression-based ranking of the numeric Cluster 1/Cluster 2 code. These heterogeneous algorithmic outputs were compared only for *post hoc* exploratory feature prioritization and were not interpreted as four independent classifiers or as estimates of classification performance.

### Single-cell RNA-seq quality control and clustering resolved the cellular landscape of GFP-control and MPL W515L model samples

To examine the cellular distribution of Fdx1 and Calr in myelofibrosis, we re-analyzed the previously published GSE228995 single-cell RNA-seq dataset derived from a murine MPL W515L-induced myelofibrosis model. The original dataset contains 22 samples spanning multiple hematopoietic and stromal populations. For the present analysis, we focused on five GFP-control and five MPL W515L model samples, designated GFP1–GFP5 and MPL1–MPL5, respectively (**Figure 6**). We next used single-cell RNA-seq to localize the candidate signal at cellular resolution. Quality-control violin plots showed the distribution of detected genes per cell, total RNA counts, and mitochondrial read percentage across GFP1-GFP5 and MPL1-MPL5 samples (**Figures 6a–c**). Detected genes and total RNA counts were strongly correlated (r = 0.84), supporting the consistency of sequencing depth and feature detection across cells (**Figure 6d**).

**Figure 6:**
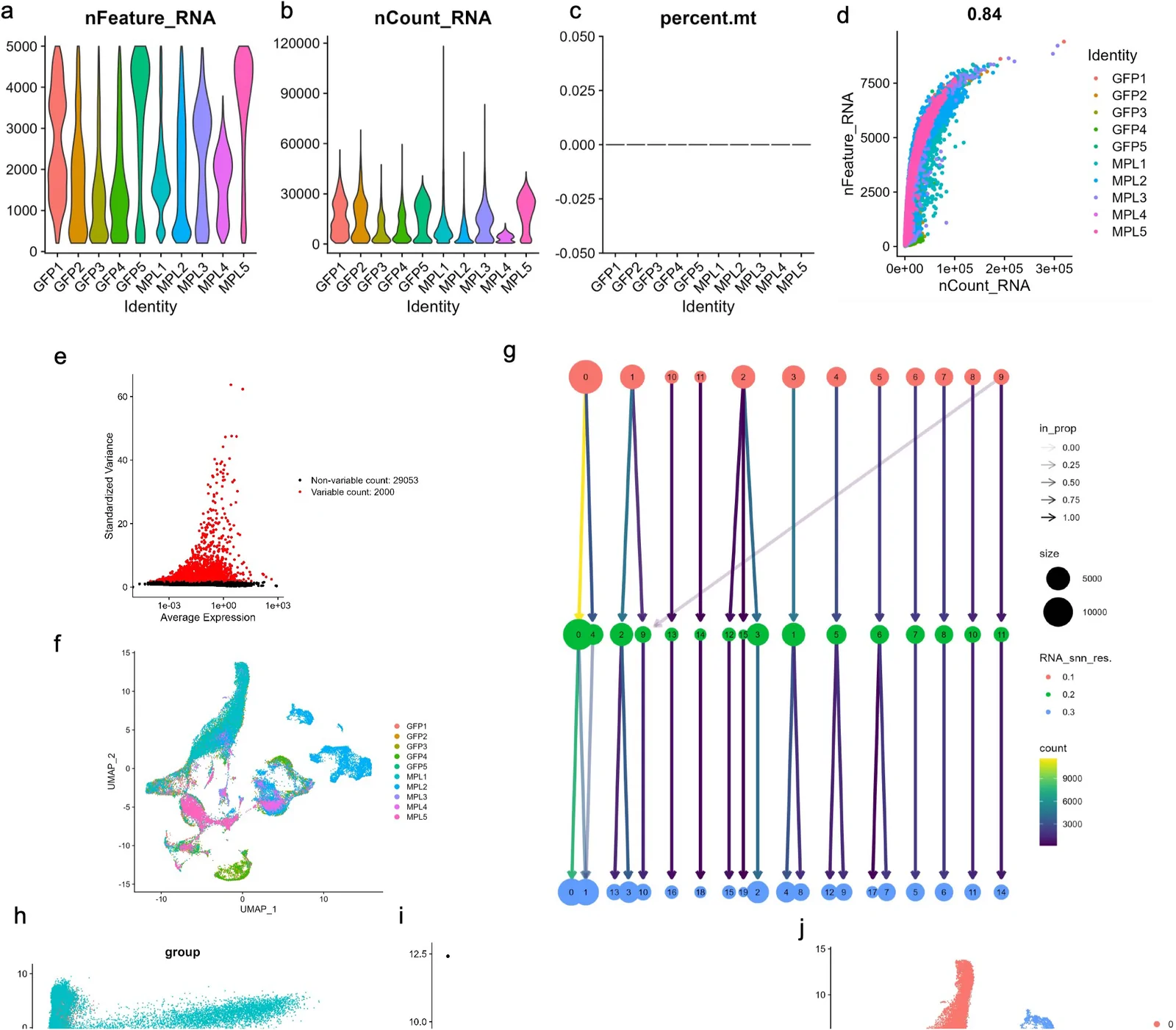
Single-cell RNA sequencing (scRNA-seq) quality control and clustering of green fluorescent protein (GFP)-control and MPL W515L model samples. **(a–c)** Violin plots showing nFeature_RNA, nCount_RNA, and mitochondrial percentage across GFP1-GFP5 and MPL1-MPL5 samples from the analyzed GSE228995 subset. **(d)** Scatter plot showing the relationship between nFeature_RNA and nCount_RNA (r = 0.84). **(e)** Highly variable gene selection, with 2,000 variable genes highlighted. **(f)** Uniform manifold approximation and projection (UMAP) visualization by sample identity. **(g)** Clustree analysis across clustering resolutions. **(h)** Principal component analysis (PCA) plot by GFP-control and MPL W515L group. **(i)** Elbow plot used to guide principal component selection. **(j)** UMAP visualization of the final 12 clusters.

After quality control, 2,000 highly variable genes were selected for downstream dimensionality reduction and clustering (**Figure 6e**). UMAP visualization by sample identity showed that GFP and MPL samples contributed to the global single-cell landscape, while some sample-specific structure was also visible (**Figure 6f**). Clustree analysis across clustering resolutions showed how clusters split across resolution parameters and supported the final clustering choice (**Figure 6g**). PCA separated cells by major transcriptional axes (**Figure 6h**), and the elbow plot was used to guide the number of principal components used for downstream analysis (**Figure 6i**). The final UMAP divided the dataset into 12 clusters, providing the framework for cell-type annotation and gene-expression mapping (**Figure 6j**).

### Calr and Fdx1 were distributed across multiple hematopoietic and immune cell populations

We annotated the single-cell clusters using canonical lineage markers. The marker dot plot showed cluster-specific expression of myeloid markers such as Mmp9, Retnlg, Mmp8, Mpo, Elane, and Ms4a3; stromal or matrix-associated markers such as Dcn, Col1a2, and Bgn; erythroid markers such as Cpox, Ermap, Klf1, Bpgm, and Alas2; B-lineage markers such as Cd79a, Cd79b, and Vpreb3; endothelial markers such as Cdh5, Ptprb, and Emcn; macrophage markers such as C1qa, C1qc, and C1qb; and megakaryocytic markers such as Pf4, Itga2b, Mmrn1, Pbx1, Clec1b, and Pld4 (**Figure 7a**). UMAP annotation resolved B cells, BM, erythroblasts, HSC_G-CSF, macrophages, monocytes, neutrophils, NK cells, Pre-B_cell_CD34-, and T cells (**Figure 7b**).

**Figure 7:**
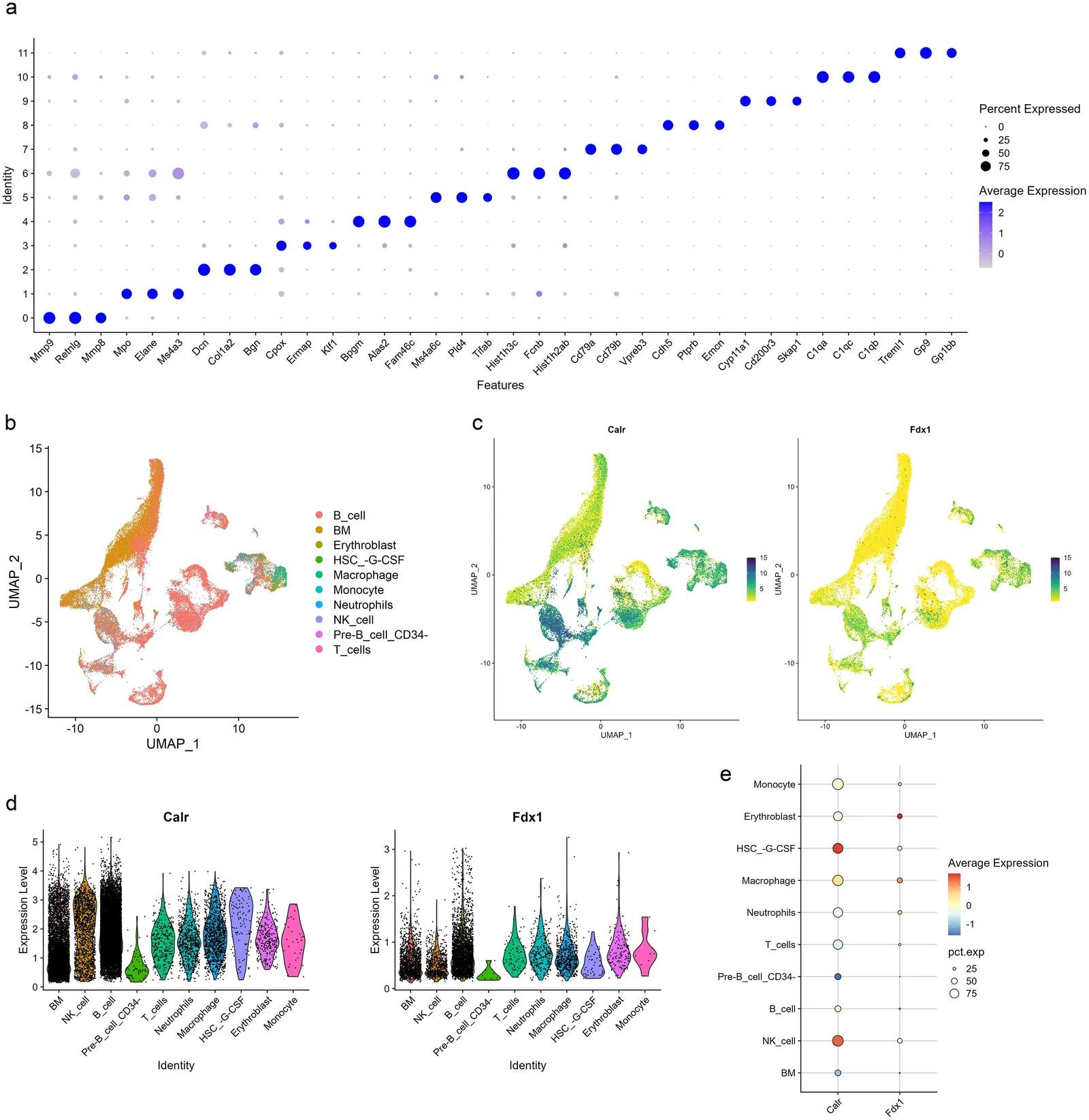
Cell-type annotation and single-cell expression of Calr and Fdx1. **(a)** Dot plot of lineage marker genes used to annotate single-cell clusters. Dot size indicates the percentage of cells expressing each marker, and color indicates average expression. **(b)** UMAP visualization of annotated cell types, including B cells, bone marrow (BM) cells, erythroblasts, HSC_G-CSF cells, macrophages, monocytes, neutrophils, natural killer (NK) cells, Pre-B_cell_CD34-, and T cells. **(c)** Feature plots showing Calr and Fdx1 expression on the UMAP map. **(d)** Violin plots showing Calr and Fdx1 expression across annotated cell types. **(e)** Dot plot summarizing Calr and Fdx1 expression and detection frequency across cell types.

Mapping the two candidate genes onto this annotated atlas showed that neither Calr nor Fdx1 was confined to one cell type. Feature plots showed broad but uneven Calr and Fdx1 expression across the UMAP, with visible signal in hematopoietic and immune compartments (**Figure 7c**). Violin plots confirmed cell-type-specific differences in expression intensity, with Calr showing strong signal in several progenitor/lymphoid-associated compartments and Fdx1 detected across erythroid, macrophage, monocyte, and other hematopoietic populations (**Figure 7d**). A summary dot plot showed both average expression and detection frequency across cell types (**Figure 7e**), confirming that Calr and Fdx1 were broadly distributed across multiple hematopoietic populations.

### Cell–cell communication analysis highlighted JAK–STAT and inflammatory ligand-receptor signaling

The single-cell atlas next allowed us to ask how annotated cell populations communicated with one another. Global communication circle plots showed dense interactions among HSC_G-CSF cells, erythroblasts, BM cells, B cells, T cells, Pre-B_cell_CD34- cells, NK cells, neutrophils, monocytes, and macrophages, with interaction number and interaction strength distributed across several cell compartments rather than dominated by one population (**Figures 8a,b**).

**Figure 8:**
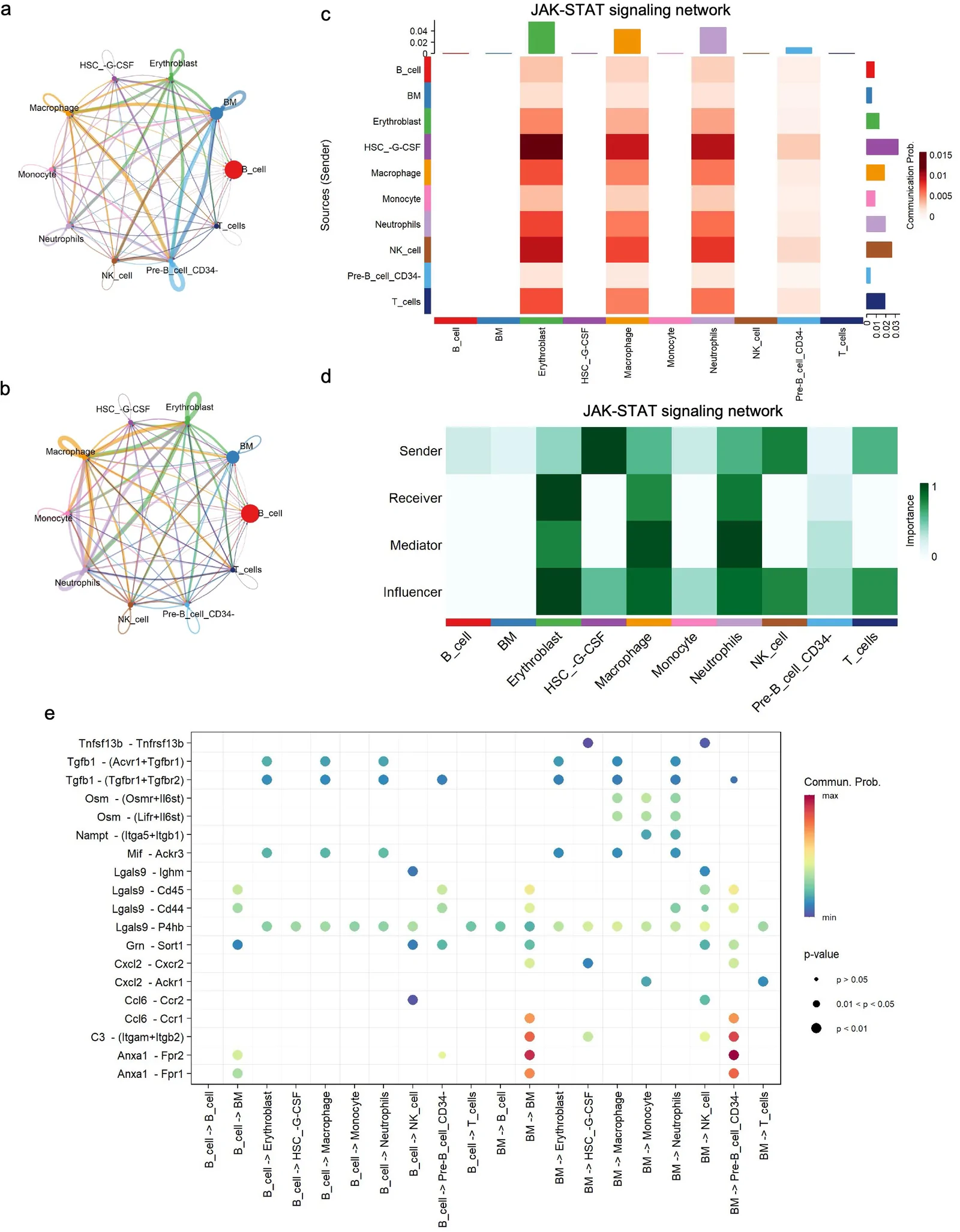
Cell-cell communication analysis highlights inferred Janus kinase-signal transducer and activator of transcription (JAK-STAT)-associated and inflammatory signaling networks. **(a)** Circle plot showing the number of inferred cell–cell interactions among annotated populations. Edge width indicates the number of significant ligand–receptor interactions. **(b)** Circle plot showing the overall inferred interaction strength among annotated populations. Edge width indicates the aggregated communication strength/probability. Node colors indicate annotated cell populations, and edge colors indicate the source/sender cell population. **(c)** Heatmap of inferred JAK–STAT-associated communication probabilities between sender and receiver cell types. **(d)** JAK–STAT network role analysis identifying sender, receiver, mediator, and influencer patterns. **(e)** Bubble plot of selected ligand-receptor interactions related to inflammatory and cytokine signaling.

Because MPN biology is tightly connected to JAK–STAT signaling, we focused on inferred JAK–STAT-associated communication. The JAK–STAT communication heatmap showed prominent sender-receiver probabilities involving HSC_G-CSF, erythroblast, macrophage, neutrophil, NK-cell, and T-cell populations (**Figure 8c**). Network-role analysis further identified HSC_G-CSF, erythroblast, macrophage, neutrophil, NK-cell, and T-cell compartments as senders, receivers, mediators, or influencers within the JAK–STAT communication network (**Figure 8d**). Bubble plots showed ligand-receptor interactions involving cytokine, inflammatory, and extracellular-matrix-associated pairs, including Tnfsf13b-Tnfrsf13b, Tgfb1 receptor complexes, Osm receptor complexes, Nampt, Mif, Lgals9, Grn, Cxcl2, Ccl6, C3, and Anxa1 (**Figure 8e**).

### Human-sample validation associated increased FDX1 and total CALR abundance with JAK–STAT-associated signaling and inflammatory output in MPN

We then validated the bioinformatic findings in human samples. Western blotting showed higher FDX1 and total CALR protein abundance in MPN samples than in controls, including lanes labeled as CALR-wild-type MPN and CALR-mutated MPN (**Figure 9a**). Because CALR-wild-type MPN may include JAK2-mutated, MPL-mutated, triple-negative, and different disease-subtype contexts, this group should be interpreted as heterogeneous. Quantification confirmed that total CALR and FDX1 protein levels were significantly increased in MPN compared with controls (P = 0.002 and P = 0.004, respectively; **Figure 9b**). Across samples, FDX1 and total CALR protein levels were positively correlated (R² = 0.33, P = 0.03), supporting an association between FDX1 and total CALR abundance rather than direct evidence of mutant CALR activity (**Figure 9c**).

**Figure 9:**
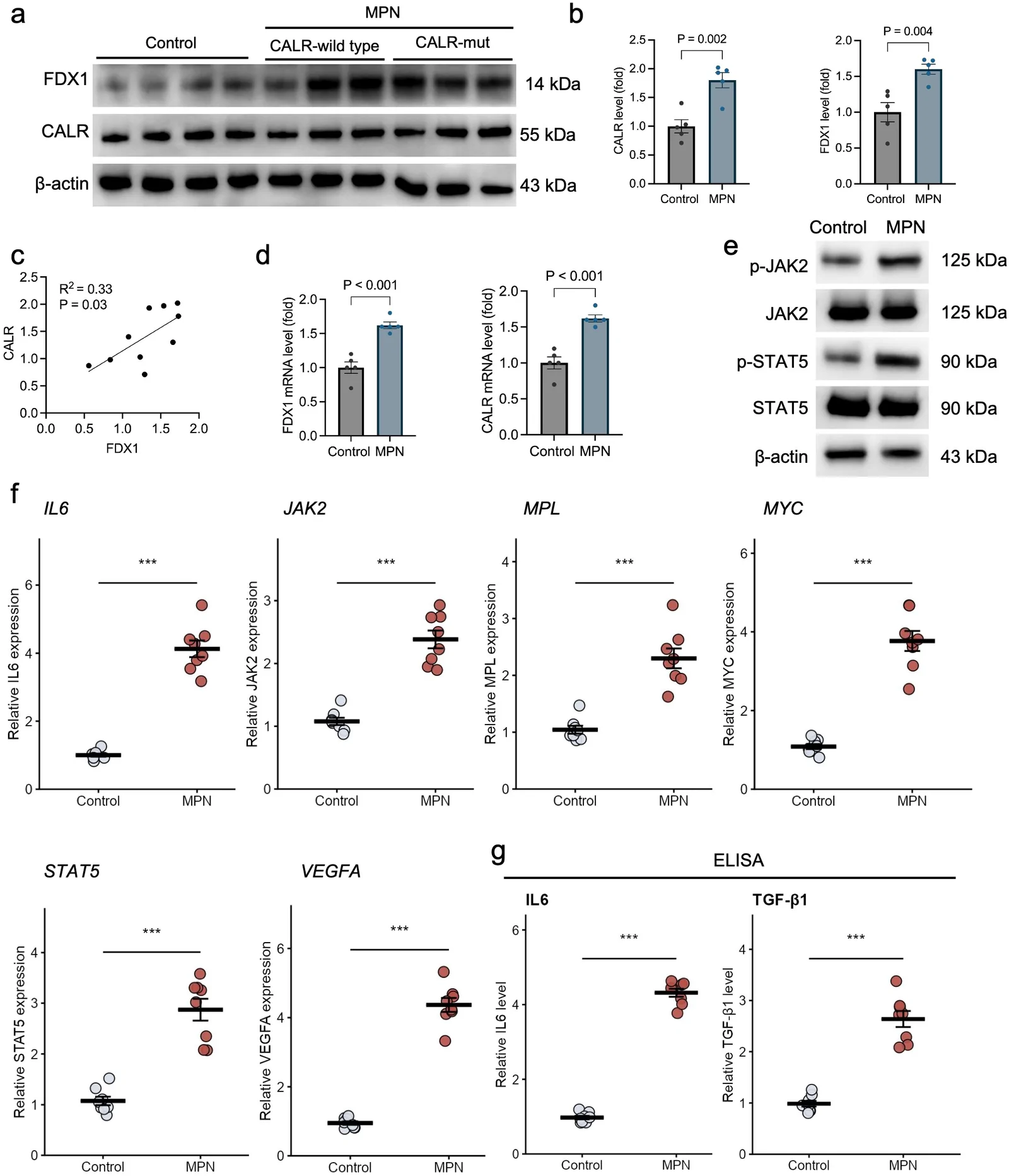
Human validation of FDX1, total CALR abundance, JAK-STAT-associated signaling, and inflammatory cytokines in MPN. **(a)** Western blots of FDX1, total CALR, and β-actin in control, CALR-wild-type MPN, and CALR-mutated MPN samples. **(b)** Quantification showing higher total CALR and FDX1 protein levels in MPN than controls. **(c)** Association between FDX1 and total CALR protein levels across samples. **(d)** Quantitative polymerase chain reaction (qPCR) validation showing increased FDX1 and CALR mRNA in MPN. **(e)** Western blots of phosphorylated JAK2 (p-JAK2), total JAK2, phosphorylated STAT5 (p-STAT5), total STAT5, and β-actin in control and MPN samples. **(f)** qPCR analysis of IL6, JAK2, MPL, MYC, STAT5, and VEGFA. **(g)** Enzyme-linked immunosorbent assay (ELISA) analysis showing increased interleukin-6 (IL-6) and transforming growth factor-β1 (TGF-β1) in MPN samples. *P < 0.05; ***P < 0.001. The asterisks displayed in panels **(f, g)** are triple asterisks (***), denoting P < 0.001.

At the RNA level, qPCR confirmed increased FDX1 and CALR mRNA in MPN samples (both P < 0.001; **Figure 9d**). JAK–STAT-associated signaling was also increased: immunoblotting showed stronger p-JAK2 and p-STAT5 signals in MPN while total JAK2 and STAT5 were used as pathway controls and β-actin as the loading control (**Figure 9e**). Consistent with pathway activation and inflammatory remodeling, qPCR showed increased IL6, JAK2, MPL, MYC, STAT5, and VEGFA expression in MPN samples (**Figure 9f**). ELISA further confirmed increased IL-6 and TGF-β1 protein levels in MPN samples (**Figure 9g**). These data support association among FDX1, total CALR abundance, JAK–STAT-associated signaling, and inflammatory cytokines in MPN.

### FDX1 perturbation modulated total CALR abundance, STAT5 phosphorylation, cytokine secretion, sensitivity to copper-dependent cytotoxicity, and proliferation

Finally, we tested whether FDX1 perturbation was associated with changes in total CALR abundance and STAT5-associated signaling. Basal western blotting showed that SET-2 and CALR-mutant cells expressed higher FDX1, total CALR, and p-STAT5 than PBMCs, with the strongest total CALR-related signal in the CALR-mutant condition (**Figure 10a**). Quantification confirmed stepwise increases in FDX1 and total CALR abundance from PBMCs to SET-2 and CALR-mutant cells (**Figure 10b**). Screening of three siRNAs showed effective FDX1 knockdown, with siFDX1-3 producing the strongest reduction and therefore used for subsequent experiments (**Figure 10c**).

**Figure 10:**
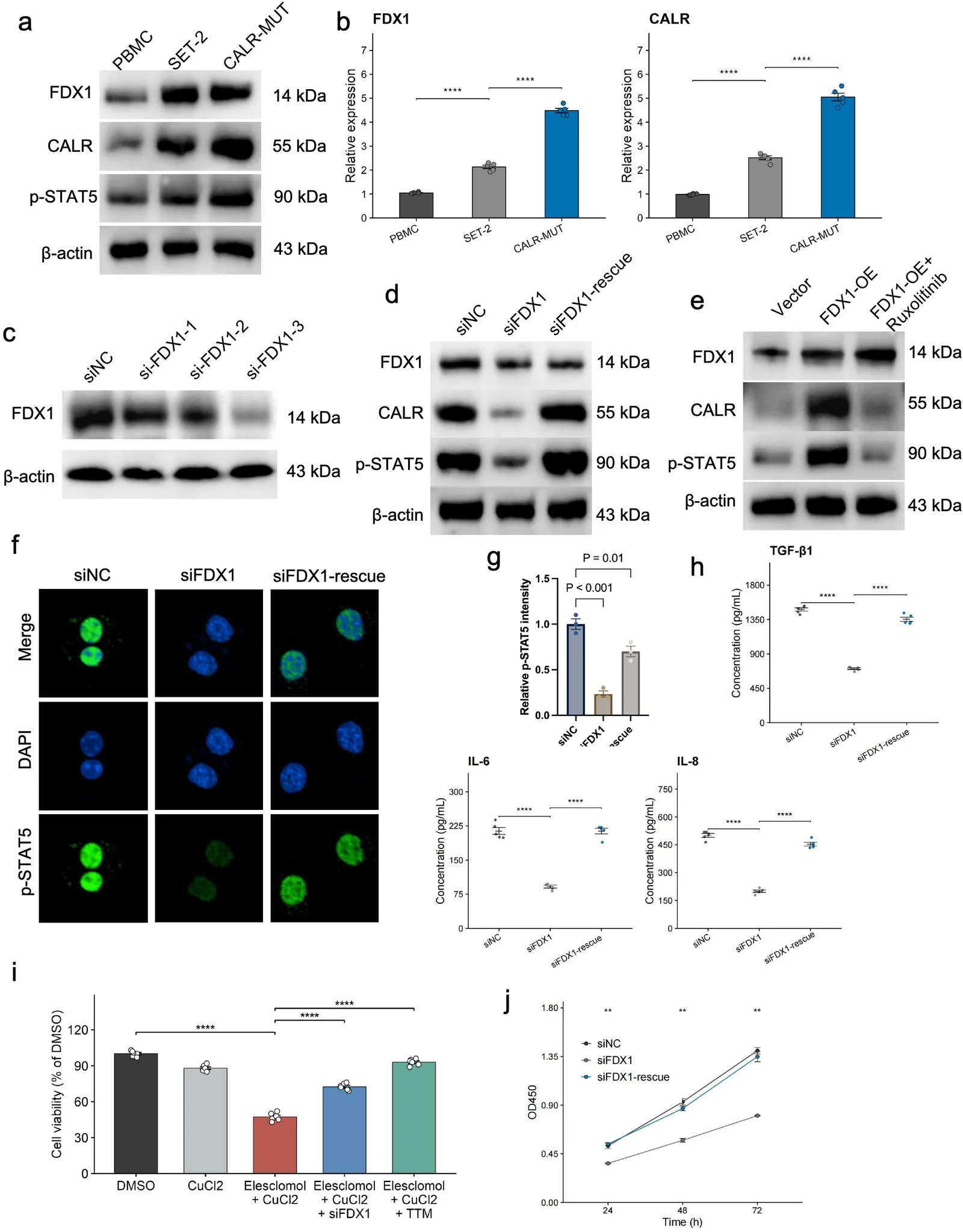
FDX1 perturbation modulates total CALR abundance, STAT5 phosphorylation, cytokine output, copper-dependent cytotoxicity, and proliferation in MPN-relevant cells. **(a)** Western blot analysis of FDX1, total CALR, p-STAT5, and β-actin in peripheral blood mononuclear cells (PBMCs), SET-2 cells, and CALR-mutant cells. **(b)** Quantification of FDX1 and total CALR abundance across PBMC, SET-2, and CALR-mutant groups. **(c)** Western blot validation of FDX1 knockdown using three small interfering RNAs (siRNAs). **(d)** Western blot showing that FDX1 knockdown decreased total CALR and p-STAT5 and that FDX1 rescue restored these signals toward control levels. **(e)** Western blot showing the effect of FDX1 overexpression and ruxolitinib treatment on total CALR and p-STAT5. **(f, g)** Immunofluorescence and quantification of p-STAT5. **(h)** Enzyme-linked immunosorbent assay (ELISA) analysis of transforming growth factor-β1 (TGF-β1), interleukin-6 (IL-6), and interleukin-8 (IL-8) after FDX1 knockdown and rescue. **(i)** Cell Counting Kit-8 (CCK-8)-based copper-dependent cytotoxicity assay with dimethyl sulfoxide (DMSO), CuCl_2_, elesclomol plus CuCl_2_, siFDX1, and tetrathiomolybdate (TTM) conditions. **(j)** CCK-8 proliferation assay at 24, 48, and 72 h in negative-control siRNA (siNC), siFDX1, and siFDX1-rescue groups. **P < 0.01; ****P < 0.0001.

FDX1 knockdown reduced total CALR abundance and p-STAT5, whereas FDX1 rescue restored both signals toward control levels, demonstrating that FDX1 perturbation is accompanied by coordinated changes in total CALR abundance and STAT5 phosphorylation in this experimental system. Because CALR detection was not allele-specific, these results do not establish direct regulation of mutant CALR or mutant CALR–MPL signaling (**Figure 10d**). Conversely, FDX1 overexpression increased FDX1, total CALR, and p-STAT5, whereas ruxolitinib suppressed p-STAT5 despite FDX1 overexpression, supporting pathway dependence at the JAK–STAT signaling level (**Figure 10e**). Immunofluorescence confirmed the western blot result: p-STAT5 staining decreased after FDX1 knockdown and increased again after FDX1 rescue (**Figures 10f,g**). These experiments do not demonstrate direct FDX1-CALR binding, direct CALR transcriptional control, or CALR protein-stability regulation.

The signaling changes were accompanied by changes in secreted cytokines and cell behavior. ELISA showed that FDX1 knockdown reduced TGF-β1, IL-6, and IL-8 secretion, whereas FDX1 rescue restored cytokine levels toward the siNC condition (**Figure 10h**). In the copper-dependent cytotoxicity assay, elesclomol plus CuCl_2_ markedly reduced cell viability compared with DMSO, while FDX1 knockdown partially rescued viability and tetrathiomolybdate (TTM) produced a stronger rescue, consistent with reduced sensitivity to copper-dependent cell death (**Figure 10i**). Proliferation assays showed reduced optical density at 450 nm (OD450) values after FDX1 knockdown and partial restoration after FDX1 rescue across 24, 48, and 72 h (**Figure 10j**). To further examine the reciprocal functional relationships among FDX1, CALR, and STAT5 signaling, reciprocal perturbation experiments were performed in CALR-mutant cells (**Supplementary Figure 4**). CALR knockdown reduced p-STAT5 without an obvious change in FDX1 or total STAT5, whereas ruxolitinib markedly suppressed p-STAT5 while FDX1, CALR, and total STAT5 remained largely unchanged. Together with the FDX1 knockdown, rescue, and overexpression experiments, these findings support an asymmetric functional relationship in which FDX1 manipulation is accompanied by changes in total CALR abundance and p-STAT5, whereas CALR depletion affects p-STAT5 without an obvious reciprocal effect on FDX1 abundance. However, these reciprocal perturbation experiments do not establish temporal ordering or demonstrate that CALR specifically mediates the effect of FDX1 on STAT5 signaling.

## Discussion

This study identifies an FDX1-associated relationship with total CALR abundance, JAK–STAT-associated signaling, inflammatory output, and copper-dependent cytotoxicity readouts in MPN. Across clinical phenotyping, microarray-based transcriptomics, exploratory machine-learning prioritization, single-cell analysis, human validation, and perturbation experiments, CALR-defined clinical features were accompanied by cuproptosis-related transcriptional changes, increased FDX1 expression, and enhanced STAT5-associated inflammatory activity. The resulting model does not replace the established mutant CALR-MPL-JAK–STAT framework; rather, it suggests that FDX1-associated cuproptosis-related metabolism may accompany or modify this signaling context. Direct biochemical interaction between FDX1 and CALR remains unproven.

The clinical observations are consistent with the known biology of CALR-mutated disease but add a metabolic and inflammatory dimension. CALR mutations were associated with thrombocytosis and megakaryocytic abnormalities, in line with the established role of mutant CALR in engaging MPL and activating JAK–STAT signaling (8, 10). However, total CALR protein abundance, CALR mutation status, mutant CALR activity, and mutant CALR-MPL signaling are distinct entities. In parallel, higher inflammatory mediators and LDH accompanied fibrotic and risk-associated features, supporting the view that MPN phenotype is shaped by both driver lesions and the surrounding inflammatory/stromal context (11–13, 25). Although increased megakaryocytic differentiation may contribute to thrombocytosis, the absence of a significant increase in megakaryocyte number in the CALR-versus-JAK2 comparison suggests that the higher platelet count cannot be explained simply by increased megakaryocyte abundance. The accompanying changes in megakaryocyte size, clustering, and nuclear morphology are more consistent with altered megakaryocyte maturation and/or platelet-producing activity. However, static bone-marrow morphology cannot distinguish increased megakaryopoietic flux from increased platelet production per megakaryocyte.

The transcriptomic analyses extend this interpretation by linking MPN disease states to cuproptosis-associated and immune-response programs. Differential expression and enrichment analyses identified genes and pathways related to inflammatory signaling, lineage activation, and metabolic remodeling, while consensus clustering separated samples into exploratory transcriptomic clusters with distinct immune and lineage features. CIBERSORT analysis further suggested that monocyte enrichment and B-lineage/plasma-cell changes may accompany the MPN-associated program, although such estimates should be interpreted as relative compositional inferences from bulk gene-expression data rather than direct cell counts (17). These findings support a model in which cuproptosis-related gene expression is embedded within broader immune remodeling rather than acting as an isolated metabolic signature.

The **Figure 5** analysis should be interpreted as *post hoc* feature prioritization rather than as a comparison of four equivalent binary classifiers. LASSO and XGBoost explicitly used binary-response objectives, whereas the random-forest and SVM-RFE workflows treated the Cluster 1/Cluster 2 label as a numeric 0/1 response and therefore generated regression-based feature rankings using %IncMSE and RMSE, respectively. Consequently, agreement among the four procedures represents recurrent prioritization across heterogeneous algorithmic formulations rather than concordant classification performance. This methodological heterogeneity, together with the outcome-informed feature space discussed above, further limits any predictive interpretation of **Figure 5**. Accordingly, these results support exploratory prioritization of FDX1 and CALR rather than diagnostic performance, a validated molecular signature, or biological causality. FDX1 is mechanistically relevant because copper-dependent cell death depends on mitochondrial metabolic context and FDX1-linked regulation (15). Its emergence in cancer-wide immune and drug-sensitivity analyses further supports the possibility that FDX1 may connect metabolic vulnerability with immune phenotype, although disease-specific validation is required in MPN (26). Importantly, although FDX1 perturbation affected both CALR/STAT5-associated signaling and sensitivity to copper-dependent cytotoxicity, our current data do not establish that these two phenotypes are mechanistically linked. Whether CALR depletion or JAK–STAT inhibition modifies FDX1-dependent copper sensitivity remains to be determined.

The single-cell data provide a cellular framework for this association. Calr and Fdx1 were distributed across multiple hematopoietic and immune populations, suggesting that the candidate program is not confined to a single annotated compartment. Cell–cell communication analysis highlighted inferred JAK–STAT-associated and inflammatory ligand-receptor interactions, which is consistent with the central role of cytokine signaling in MPN biology and with prior single-cell studies showing that clonal identity, lineage bias, and inflammatory programs can be resolved at single-cell resolution in myeloid neoplasia (27, 28). Because CellChat infers communication potential from ligand and receptor expression rather than measuring physical interactions, these results should be interpreted as hypothesis-generating maps of signaling architecture (16).

The validation and perturbation experiments move the study from association toward functional testing, but the mechanistic boundary remains important. Human MPN samples showed increased FDX1 and total CALR expression together with STAT5 phosphorylation and increased inflammatory mediators. In cellular experiments, FDX1 knockdown reduced total CALR abundance, STAT5 phosphorylation, cytokine secretion, and proliferative output, whereas FDX1 overexpression produced the reciprocal phenotype and ruxolitinib partly counteracted the p-STAT5-associated response. Because ruxolitinib clinically targets JAK1/2 signaling and improves key disease manifestations in myelofibrosis (14), this pharmacological result places the FDX1-associated phenotype within a therapeutically relevant JAK–STAT signaling context. The reciprocal perturbation experiments further support an asymmetric functional relationship: CALR depletion reduced p-STAT5 without an obvious change in FDX1, whereas JAK inhibition reduced p-STAT5 without substantially altering FDX1 or total CALR abundance. However, because temporal ordering was not examined and no epistasis experiment directly tested whether CALR depletion prevents the STAT5-associated effects of FDX1 manipulation, these data do not establish CALR as a necessary mediator between FDX1 and STAT5 signaling. An important unresolved question is whether the association between FDX1 and total CALR abundance reflects direct regulation or an indirect consequence of metabolic and cellular stress. FDX1 is a mitochondrial ferredoxin that regulates protein lipoylation and copper-dependent metabolic responses (15). FDX1 can directly interact with LIAS, and FDX1 loss alters mitochondrial respiration and cellular metabolism while inducing an integrated stress response (29). In contrast, CALR is an ER-resident chaperone whose expression is responsive to cellular proteostasis and ER stress; notably, ATF6-dependent UPR activation can increase CALR expression in myeloid leukemia cells. CALR-mutated MPN cells also exhibit altered proteostasis and ER-stress programs (30). These observations raise the possibility that the changes in total CALR abundance following FDX1 perturbation arise indirectly from mitochondrial metabolic remodeling, cellular stress, or inflammatory signaling. Our present data cannot distinguish these possibilities from direct transcriptional, translational, or protein-stability regulation of CALR by FDX1, and direct biochemical interaction between FDX1 and CALR remains to be established.

Several limitations define the boundary of this interpretation. An additional limitation concerns the *post hoc* feature-prioritization analysis. Because the transcriptomic cluster labels were derived from the same expression matrix used to define Cluster 1-versus-Cluster 2 DEGs, the DEG-derived feature space was outcome-informed before subsequent algorithmic feature prioritization. This analytical circularity limits independent interpretation of the algorithmic overlap. Formal biomarker discovery or predictive validation would require a larger independent cohort with prespecified outcomes, classification-appropriate model implementation, and a fully nested feature-selection and validation framework. The clinical cohort is relatively limited in size and includes heterogeneous MPN subtypes, driver mutations, fibrosis grades, disease stages, and treatment backgrounds, all of which may influence molecular and inflammatory profiles and potentially confound the observed associations. Although the current 60-case cohort represents the largest cohort available to us under the present study conditions, validation in a larger and independent cohort is warranted. Future multicenter studies with more balanced subtype representation and standardized clinical and molecular annotation will be important for subtype-stratified and multivariable validation of the associations among FDX1, total CALR abundance, and JAK–STAT-related inflammatory signaling. CALR-versus-non-CALR comparisons may be confounded by disease subtype, fibrosis grade, age, sex, treatment, and other driver mutations. GSE174060 is a microarray dataset rather than an RNA-sequencing dataset, and all differential-expression and downstream analyses should therefore be interpreted within a microarray-analysis framework. Bulk transcriptomic and immune-deconvolution analyses are sensitive to cohort composition and should be validated by orthogonal cell-counting and spatial methods. The GSE228995 single-cell dataset derives from a murine MPL W515L-induced myelofibrosis model and may not fully represent human CALR-mutated disease. The single-cell analysis did not directly assign every cell to a mutation-defined clone; allele-aware single-cell genotyping would clarify whether FDX1-high states are preferentially enriched in CALR-mutant hematopoiesis. The relationship between FDX1 and copper-dependent cell death also requires careful interpretation. Cuproptosis is mechanistically defined by copper binding to lipoylated mitochondrial TCA-cycle proteins, resulting in lipoylated protein aggregation, Fe–S cluster protein loss, proteotoxic stress, and cell death that is distinct from apoptosis, ferroptosis, and necroptosis (15). Accordingly, altered FDX1 expression or sensitivity to copper ionophores alone is not sufficient to establish canonical cuproptosis. FDX1 has broader functions in mitochondrial metabolism: it directly interacts with LIAS to regulate protein lipoylation, and FDX1 loss alters mitochondrial respiration, metabolic homeostasis, and the integrated stress response (29). Moreover, FDX1 facilitates mitochondrial release of copper from the elesclomol–Cu complex, although FDX1-independent copper-delivery mechanisms also exist (31). Therefore, the reduced sensitivity to elesclomol/CuCl_2_ after FDX1 knockdown and its rescue by copper chelation in our experiments support an FDX1-associated role in copper-dependent cytotoxicity, but do not establish that canonical cuproptosis is the sole or dominant mechanism. Direct assessment of lipoylated DLAT aggregation, Fe–S cluster protein loss, intracellular copper accumulation, and exclusion of alternative regulated cell-death pathways will be required to define this mechanism more precisely in future studies. Finally, canonical endogenous cuproptosis markers, such as lipoylated DLAT aggregation, Fe-S cluster protein loss, and exclusion of other death programs, were not documented. Future studies should test whether FDX1 modulates total CALR expression through transcriptional, translational, proteostatic, or indirect stress-response mechanisms and whether this relationship creates a therapeutic vulnerability in MPN.

## Data Availability

The original contributions presented in the study are included in the article/**Supplementary Material**, further inquiries can be directed to the corresponding author/s.

## References

[1] Khoury JD Solary E Abla O Akkari Y Alaggio R Apperley JF . The 5th edition of the World Health Organization classification of haematolymphoid tumours: Myeloid and histiocytic/dendritic neoplasms. Leukemia. (2022) 36:1703–19. doi: 10.1038/s41375-022-01613-1 35732831 PMC9252913

[2] Arber DA Orazi A Hasserjian RP Borowitz MJ Calvo KR Kvasnicka HM . International consensus classification of myeloid neoplasms and acute leukemias: Integrating morphologic, clinical, and genomic data. Blood. (2022) 140:1200–28. doi: 10.1182/blood.2022015850 35767897 PMC9479031

[3] Nangalia J Massie CE Baxter EJ Nice FL Gundem G Wedge DC . Somatic CALR mutations in myeloproliferative neoplasms with nonmutated JAK2. N Engl J Med. (2013) 369:2391–405. doi: 10.1056/NEJMoa1312542 24325359 PMC3966280

[4] Klampfl T Gisslinger H Harutyunyan AS Nivarthi H Rumi E Milosevic JD . Somatic mutations of calreticulin in myeloproliferative neoplasms. N Engl J Med. (2013) 369:2379–90. doi: 10.1056/nejmoa1311347 24325356

[5] Kralovics R Passamonti F Buser AS Teo SS Tiedt R Passweg JR . A gain-of-function mutation of JAK2 in myeloproliferative disorders. N Engl J Med. (2005) 352:1779–90. doi: 10.1056/nejmoa051113 15858187

[6] Baxter EJ Scott LM Campbell PJ East C Fourouclas N Swanton S . Acquired mutation of the tyrosine kinase JAK2 in human myeloproliferative disorders. Lancet. (2005) 365:1054–61. doi: 10.1016/s0140-6736(05)71142-9 15781101

[7] James C Ugo V Le Couédic JP Staerk J Delhommeau F Lacout C . A unique clonal JAK2 mutation leading to constitutive signalling causes polycythaemia vera. Nature. (2005) 434:1144–8. doi: 10.1038/nature03546 15793561

[8] Elf S Abdelfattah NS Chen E Perales-Patón J Rosen EA Ko A . Mutant calreticulin requires both its mutant C-terminus and the thrombopoietin receptor for oncogenic transformation. Cancer Discov. (2016) 6:368–81. doi: 10.1158/2159-8290.cd-15-1434 26951227 PMC4851866

[9] Chachoua I Pecquet C El-Khoury M Nivarthi H Albu RI Marty C . Thrombopoietin receptor activation by myeloproliferative neoplasm associated calreticulin mutants. Blood. (2016) 127:1325–35. doi: 10.1182/blood-2015-11-681932 26668133

[10] Stanley RF Steidl U . Molecular mechanism of mutant CALR-mediated transformation. Cancer Discov. (2016) 6:344–6. doi: 10.1158/2159-8290.cd-16-0238 27045016 PMC4832981

[11] Koschmieder S Mughal TI Hasselbalch HC Barosi G Valent P Kiladjian JJ . Myeloproliferative neoplasms and inflammation: Whether to target the Malignant clone or the inflammatory process or both. Leukemia. (2016) 30:1018–24. doi: 10.1038/leu.2016.12 26854026

[12] Hasselbalch HC . The role of cytokines in the initiation and progression of myelofibrosis. Cytokine Growth Factor Rev. (2013) 24:133–45. doi: 10.1016/j.cytogfr.2013.01.004 23415024

[13] Koschmieder S Chatain N . Role of inflammation in the biology of myeloproliferative neoplasms. Blood Rev. (2020) 42:100711. doi: 10.1016/j.blre.2020.100711 32505517

[14] Verstovsek S Mesa RA Gotlib J Levy RS Gupta V DiPersio JF . A double-blind, placebo-controlled trial of ruxolitinib for myelofibrosis. N Engl J Med. (2012) 366:799–807. doi: 10.1056/nejmoa1110557 22375971 PMC4822164

[15] Tsvetkov P Coy S Petrova B Dreishpoon M Verma A Abdusamad M . Copper induces cell death by targeting lipoylated TCA cycle proteins. Science. (2022) 375:1254–61. doi: 10.1126/science.abf0529 35298263 PMC9273333

[16] Jin S Guerrero-Juarez CF Zhang L Chang I Ramos R Kuan CH . Inference and analysis of cell-cell communication using CellChat. Nat Commun. (2021) 12:1088. doi: 10.1038/s41467-021-21246-9 33597522 PMC7889871

[17] Newman AM Liu CL Green MR Gentles AJ Feng W Xu Y . Robust enumeration of cell subsets from tissue expression profiles. Nat Methods. (2015) 12:453–7. doi: 10.1038/nmeth.3337 25822800 PMC4739640

[18] Baumeister J Maié T Chatain N Gan L Weinbergerova B de Toledo MAS . Early and late stage MPN patients show distinct gene expression profiles in CD34+ cells. Ann Hematol. (2021) 100:2943–56. doi: 10.1007/s00277-021-04615-8 34390367 PMC8592960

[19] Smyth GK . Linear models and empirical bayes methods for assessing differential expression in microarray experiments. Stat Appl Genet Mol Biol. (2004) 3:Article3. doi: 10.2202/1544-6115.1027 16646809

[20] Yu G Wang L-G Han Y He Q-Y . clusterProfiler: An R package for comparing biological themes among gene clusters. Omics J Integr Biol. (2012) 16:284–7. doi: 10.1089/omi.2011.0118 22455463 PMC3339379

[21] Wilkerson MD Hayes DN . ConsensusClusterPlus: A class discovery tool with confidence assessments and item tracking. Bioinformatics. (2010) 26:1572–3. doi: 10.1093/bioinformatics/btq170 20427518 PMC2881355

[22] Friedman J Hastie T Tibshirani R . Regularization paths for generalized linear models via coordinate descent. J Stat Softw. (2010) 33:1–22. doi: 10.18637/jss.v033.i01 PMC2929880 20808728

[23] Li R Colombo M Wang G Rodriguez-Romera A Benlabiod C Jooss NJ . A proinflammatory stem cell niche drives myelofibrosis through a targetable galectin-1 axis. Sci Transl Med. (2024) 16:eadj7552. doi: 10.1126/scitranslmed.adj7552 39383242 PMC7616771

[24] Hao Y Hao S Andersen-Nissen E Mauck WM Zheng S Butler A . Integrated analysis of multimodal single-cell data. Cell. (2021) 184:3573–3587.e29. doi: 10.1016/j.cell.2021.04.048 34062119 PMC8238499

[25] Tefferi A Vaidya R Caramazza D Finke C Lasho T Pardanani A . Circulating interleukin (IL)-8, IL-2R, IL-12, and IL-15 levels are independently prognostic in primary myelofibrosis: A comprehensive cytokine profiling study. J Clin Oncol Off J Am Soc Clin Oncol. (2011) 29:1356–63. doi: 10.1200/jco.2010.32.9490 21300928

[26] Yang L Zhang Y Wang Y Jiang P Liu F Feng N . Ferredoxin 1 is a cuproptosis-key gene responsible for tumor immunity and drug sensitivity: A pan-cancer analysis. Front Pharmacol. (2022) 13:938134. doi: 10.3389/fphar.2022.938134 36210836 PMC9532935

[27] Nam AS Kim KT Chaligne R Izzo F Ang C Taylor J . Somatic mutations and cell identity linked by genotyping of transcriptomes. Nature. (2019) 571:355–60. doi: 10.1038/s41586-019-1367-0 31270458 PMC6782071

[28] Psaila B Wang G Rodriguez-Meira A Li R Heuston EF Murphy L . Single-cell analyses reveal megakaryocyte-biased hematopoiesis in myelofibrosis and identify mutant clone-specific targets. Mol Cell. (2020) 78:477–492.e8. doi: 10.1016/j.molcel.2020.04.008 32386542 PMC7217381

[29] Dreishpoon MB Bick NR Petrova B Warui DM Cameron A Booker SJ . FDX1 regulates cellular protein lipoylation through direct binding to LIAS. J Biol Chem. (2023) 299:105046. doi: 10.1016/j.jbc.2023.105046 37453661 PMC10462841

[30] Jutzi JS Marneth AE Jiménez-Santos MJ Hem J Guerra-Moreno A Rolles B . CALR-mutated cells are vulnerable to combined inhibition of the proteasome and the endoplasmic reticulum stress response. Leukemia. (2023) 37:359–69. doi: 10.1038/s41375-022-01781-0 36473980 PMC12413246

[31] Zulkifli M Spelbring AN Zhang Y Soma S Chen S Li L . FDX1-dependent and independent mechanisms of elesclomol-mediated intracellular copper delivery. Proc Natl Acad Sci USA. (2023) 120:e2216722120. doi: 10.1073/pnas.2216722120 36848556 PMC10013847

