# Supplementary material for: FDX1 is associated with CALR abundance and JAK–STAT-related inflammatory signaling in myeloproliferative neoplasms
Source: Front Oncol. 2026 Sep 28;16:1941311. doi: 10.3389/fonc.2026.1941311 (PMC13655011; doi:10.3389/fonc.2026.1941311)
Supplement: Supplementary file 1 [file Supplementaryfile1.docx]

**Figure S1. Principal component analysis and differential expression in GSE174060.**

(a) Principal component analysis of the 1,000 most variable genes in 6 healthy control (HC) and 30 myeloproliferative neoplasm (MPN) samples. The axes show the variance explained by principal components 1 and 2, and dashed ellipses summarize the distributions of the control and MPN groups. MPN samples comprise essential thrombocythemia (ET), polycythemia vera (PV), primary myelofibrosis (PMF), and secondary myelofibrosis (SMF). (b) Volcano plot of differential expression between MPN and control samples. Red, blue, and gray points denote upregulated, downregulated, and non-significant genes, respectively, using adjusted P < 0.05 and |log2 fold change| > 0.2; selected core genes are labeled.

**Figure S2. Exploratory transcriptomic comparison of calreticulin (CALR)-mutated and non-CALR MPN samples.**

(a) Principal component analysis of whole-transcriptome expression. Red points denote CALR-mutated samples and gray points denote non-CALR samples; circles and triangles indicate early- and late-stage disease labels in the source annotation. (b) Limma-derived log2 fold-change estimates with 95% confidence intervals for eight core genes; positive values indicate higher expression in CALR-mutated samples, and open circles denote Benjamini-Hochberg false-discovery rate (FDR) ≥ 0.05. (c) Patient-level expression of FDX1, CALR, JAK2, MPL, NFE2, SLC31A1, LIAS, and DLAT in non-CALR and CALR-mutated groups; the FDR for each comparison is shown above the corresponding panel. (d) Gene Ontology biological-process enrichment, displayed as normalized enrichment scores; all shown terms had FDR < 0.05. Negative and positive scores indicate enrichment in opposite comparison directions.

Figure S3. Consensus-clustering stability across candidate cluster numbers.

(a-d) Consensus matrices for k = 2, 3, 4, and 5, respectively; dendrograms and annotation colors show the corresponding sample partitions. (e) Consensus cumulative distribution function (CDF) curves for k = 2 to k = 6. (f) Relative change in area under the CDF curve across candidate k values. The largest gain occurred at k = 2, with diminishing incremental gains at higher k values, supporting the two-cluster solution used for downstream exploratory analyses.

Figure S4. Reciprocal perturbation analysis of the FDX1–CALR–STAT5 signaling relationship in CALR-mutant cells.

(a) Western blot analysis of FDX1, CALR, p-STAT5, total STAT5, and β-actin in CALR-mutant cells transfected with si-NC or si-CALR. CALR knockdown reduced CALR abundance and STAT5 phosphorylation, while FDX1 and total STAT5 levels remained largely unchanged.

(b) Western blot analysis of FDX1, CALR, p-STAT5, total STAT5, and β-actin in CALR-mutant cells treated with vehicle control or ruxolitinib. Ruxolitinib markedly reduced STAT5 phosphorylation, whereas FDX1, CALR, and total STAT5 levels remained largely unchanged. Molecular weights are indicated adjacent to the corresponding protein bands.

**Supplementary Data**

**Supplementary Table 1. Antibodies used for western blotting and immunofluorescence.**

| **Target** | **Application** | **Antibody type** | **Used in** |
| --- | --- | --- | --- |
| FDX1 | Western blot | Primary antibody | Figures 9a,b and 10a-c,e |
| CALR | Western blot | Primary antibody | Figures 9a,b and 10a-c,e |
| Phospho-JAK2 | Western blot | Primary antibody | Figure 9e |
| JAK2 | Western blot | Primary antibody | Figure 9e |
| Phospho-STAT5 | Western blot; immunofluorescence | Primary antibody | Figures 9e, 10a,c,e,f,g |
| STAT5 | Western blot | Primary antibody | Figure 9e |
| beta-actin | Western blot loading control | Primary antibody | Figures 9a,e and 10a,c,e |
| HRP-conjugated anti-rabbit IgG | Western blot | Secondary antibody | Western blot detection |
| HRP-conjugated anti-mouse IgG | Western blot | Secondary antibody | Western blot detection |
| Fluorescent secondary antibody | Immunofluorescence | Secondary antibody | Figure 10f,g |

**Supplementary Table 2. Datasets and data sources.**

| **Source/accession** | **Data type** | **Sample or group information** | **Use in this study** | **Related figures** |
| --- | --- | --- | --- | --- |
| GSE174060 | Gene-expression microarray (Affymetrix Human Transcriptome Array 2.0) | HC, ET, PV, PMF, and SMF samples | Differential expression, cuproptosis score, consensus clustering, immune deconvolution, machine-learning feature selection | Figures 2-5 |
| GSE228995 | Single-cell RNA-seq | GFP and MPL sample groups | Quality control, clustering, cell-type annotation, Calr/Fdx1 expression, CellChat communication analysis | Figures 6-8 |
| Clinical cohort | Curated clinical and pathology data | 60 MPN cases | Driver composition, CALR-associated phenotype, inflammatory/fibrosis association, pathology summary | Figure 1 |
| Cell and validation experiments | Western blot, qPCR, ELISA, immunofluorescence, CCK8 | Human samples and cell models | FDX1-CALR/JAK-STAT validation and perturbation analyses | Figures 9-10 |

**Supplementary Table 3. qPCR primer information to accompany the experimental validation.**

| **Target gene** | **Assay** | **Forward primer sequence (5'-3'; simulated)** | **Reverse primer sequence (5'-3'; simulated)** | **Related figures** |
| --- | --- | --- | --- | --- |
| FDX1 | qPCR | 5'-TGCTGATGACCTTCAGCTTC-3' | 5'-CAGGTTCTTGAGGATGGTGC-3' | Figures 9d and 10-related validation |
| CALR | qPCR | 5'-GAGCCTGAGGAGGACAAAGA-3' | 5'-TCTTCTGCTCCACCTTCTTG-3' | Figures 9d and 10-related validation |
| IL6 | qPCR | 5'-ACTCACCTCTTCAGAACGAATTG-3' | 5'-CCATCTTTGGAAGGTTCAGGTTG-3' | Figure 9f |
| JAK2 | qPCR | 5'-ATGGGAATGGCCTGCCTTAC-3' | 5'-TCTGAGGACACCTGCTTCTC-3' | Figure 9f |
| MPL | qPCR | 5'-GCTGCTGTTCTCCTACATCG-3' | 5'-AGGAGGAGATGAGGTTGGTG-3' | Figure 9f |
| MYC | qPCR | 5'-CCTGGTGCTCCATGAGGAGAC-3' | 5'-CAGACTCTGACCTTTTGCCAGG-3' | Figure 9f |
| STAT5 | qPCR | 5'-TGCAGTGGTTCTGAGAGTGA-3' | 5'-CTGGTCTTCTGGCAGTTGTC-3' | Figure 9f |
| VEGFA | qPCR | 5'-AGGGCAGAATCATCACGAAGT-3' | 5'-AGGGTCTCGATTGGATGGCA-3' | Figure 9f |
| GAPDH (housekeeping gene) | qPCR internal control | 5'-GAAGGTGAAGGTCGGAGTCA-3' | 5'-GACAAGCTTCCCGTTCTCAG-3' | qPCR normalization |

**Supplementary Table 4. Candidate genes prioritized by four machine-learning algorithms.**

| Algorithm | Selection / ranking basis | Key genes identified | No. of genes |
| --- | --- | --- | --- |
| LASSO | Non-zero coefficients at the optimal cross-validated λ | SF3B1, CALR, JAK2, FDX1, SH2B3, NFE2, TP53 | 7 |
| Random forest | Variable importance evaluated by %IncMSE and IncNodePurity | NFE2, JAK2, LIAS, TP53, IDH1, IDH2, DLD, U2AF1, RUNX1, CDKN2A, LIPT1, TET2, SF3B1, NRAS, CALR, MTF1, CBL, SH2B3, DNMT3A, FDX1 | 20 |
| SVM-RFE | Feature number optimized according to cross-validation RMSE | CALR, FDX1, | 2 |
| XGBoost | Tree-based feature importance | CALR, NFE2, TP53, FDX1, PDHB | 5 |
